# Diversity, Distribution, Systematics and Conservation Status of Podocarpaceae

**DOI:** 10.3390/plants12051171

**Published:** 2023-03-03

**Authors:** Raees Khan, Robert S. Hill, Jie Liu, Ed Biffin

**Affiliations:** 1 School of Biological Sciences, The University of Adelaide, Adelaide, SA 5005, Australia; 2 CAS Key Laboratory for Plant Diversity and Biogeography of East Asia, Kunming Institute of Botany, Chinese Academy of Sciences, Kunming 650201, China; 3 Germplasm Bank of Wild Species, Kunming Institute of Botany, Chinese Academy of Sciences, Kunming 650201, China

**Keywords:** conservation, conifers, climate change, fossils, historical biogeography, IUCN red list, paleo-endemism, physiology, phylogenetics

## Abstract

Among conifer families, Podocarpaceae is the second largest, with amazing diversity and functional traits, and it is the dominant Southern Hemisphere conifer family. However, comprehensive studies on diversity, distribution, systematic and ecophysiological aspects of the Podocarpaceae are sparse. We aim to outline and evaluate the current and past diversity, distribution, systematics, ecophysiological adaptations, endemism, and conservation status of podocarps. We analyzed data on the diversity and distribution of living and extinct macrofossil taxa and combined it with genetic data to reconstruct an updated phylogeny and understand historical biogeography. Podocarpaceae today contains 20 genera and approximately 219 taxa (201 species, 2 subspecies, 14 varieties and 2 hybrids) placed in three clades, plus a paraphyletic group/grade of four distinct genera. Macrofossil records show the presence of more than 100 podocarp taxa globally, dominantly from the Eocene–Miocene. Australasia (New Caledonia, Tasmania, New Zealand, and Malesia) is the hotspot of living podocarps diversity. Podocarps also show remarkable adaptations from broad to scale leaves, fleshy seed cones, animal dispersal, shrubs to large trees, from lowland to alpine regions and rheophyte to a parasite (including the only parasitic gymnosperm—Parasitaxus) and a complex pattern of seed and leaf functional trait evolution.

## 1. Introduction

Conifers are economically and ecologically important, form extensive forests in both Hemispheres and are currently the most diverse gymnosperms. There are seven conifer families (Araucariaceae, Cupressaceae, Pinaceae, Podocarpaceae, Sciadopityaceae, Cephalotaxaceae and Taxaceae), including 72 genera and approximately 702 species [1]. They are estimated to have evolved in the late Devonian from progymnosperms, and then dominated the Mesozoic Era [2,3,4]. Leslie et al. [5] investigated the evolutionary dynamics of conifers on a hemispheric scale based on molecular studies of 489 species and concluded that extant conifers have diverged in the Neogene with older splits in the Southern Hemisphere. Pinaceae and Cupressaceae have their main distribution in the temperate and subtropical regions of the Northern Hemisphere, while the Southern Hemisphere conifers are dominated by the Araucariaceae, Podocarpaceae and the Callitroideae of the Cupressaceae. The podocarps are monoecious and dioecious evergreen trees, shrubs, and subshrubs with mostly spirally arranged leaves and fleshy cones [6]. They are morphologically highly diverse [7,8]. Although the ecological and environmental variation (mostly rainforest and wet montane) is restricted, the morphological variation in leaves and seed cones is very high [9,10]. The extant and extinct taxa present in Australasia and South America show the wider distribution of the family across Gondwana in the past. Phylogenetically, the Podocarpaceae are closest to the Araucariaceae, Sciadopityaceae and Taxaceae [11,12]. Podocarps are significant both ecologically and economically and are a vital component of global forests and biodiversity [4,13]. The Podocarpaceae are very important from an evolutionary and systematic point of view due to their remarkable eco-physiological adaptations as compared to other conifer families, and they can provide us with valuable information on evolution and response to climate change [4,14]. Podocarps provide an exceptional opportunity for the understanding of comparative diversification processes, the evolution of different functional traits and ecophysiological adaptations. However, comprehensive studies are lacking on the different taxonomic, phylogenetic, ecological, biogeographic, and evolutionary aspects of most Podocarpaceae due to fragmented data on living species, sparse fossil records, difficulty in sampling and ecological data collection and less attraction compared to other conifer families. Many species described from Papua New Guinea, Malaysia, Indonesia, and New Caledonia are based on single records, and these areas remain under-explored. To evaluate these different aspects and several other key features, updated checklists for living and extinct taxa, phylogenetic analysis and ecophysiological adaptation research is required.

In this study, we evaluate the diversity, distribution, taxonomy, phylogeny, ecophysiology, and ecology of podocarps with the following aims: 1. To tabulate updated podocarp checklists for both extant and extinct taxa. 2. To reconstruct a new dated phylogeny of Podocarpaceae using relevant macrofossil records. 3. To assess the historical overview of taxonomic classifications of podocarps. 4. To discuss the diversity and historical biogeography. 5. To consider ecophysiological adaptations and threats.

## 2. Material and Methods

### 2.1. Phylogenetic Studies

A new, dated phylogenetic tree was produced for the Podocarpaceae. For phylogenetic analysis, DNA sequences of Podocarpaceae and Araucariaceae were sourced from GenBank (https://www.ncbi.nlm.nih.gov/genbank/, accessed on 25 January 2021). The sequences were cleaned and six markers, *rbcL*, *matK*, *trnL*-*trnF*, NEEDLY, PHYP and ITS species, were selected based on alignment confidence and availability of sequences. The final concatenated alignment consists of 190 taxa with 14 taxa as an out-group. The data were analyzed with BEAST version 2.6.3 at the CIPRES Science Gateway [15], set to run an uncorrelated lognormal relaxed clock and a GTR+I+G substitution model [16]. For calibration of the tree, we used the 20 oldest unequivocal macrofossil records (see supplementary file Table S1). Sixteen fossil constraints were assigned to the Podocarpaceae, two were assigned to the Araucariaceae and two were assigned to other conifers. The Fossil Birth–Death (FBD) model [17] was used as the tree prior, which imposes a time structure on the tree, while accounting for uncertainty in the placement of the fossil data by allowing all plausible placements for the fossil taxon on the extant tree [18].

### 2.2. Updated Checklist, Distribution, and IUCN Conservation Status of Podocarps

An updated checklist of all podocarp species was compiled based on the available literature [4], herbarium specimen observations and online databases, e.g., The Gymnosperm Database [19]; Global Biodiversity Information Facility-GBIF [20]; plants of the world online [21]; Australasian Virtual Herbarium-AVH [22]; Flora of China [23]. 

### 2.3. Distribution Data Analysis

The distribution of the species was analyzed in PC-ORD [24]. The Cluster Analysis (CA) and the Two-Way Cluster Analyses (TWCA) were used to identify significant and species-rich countries using Sorensen measures, based on presence/absence data.

### 2.4. Updated Checklist for Macrofossils of Extant Genera of Podocarpaceae

A checklist of fossil podocarps species belonging to extant genera was compiled using published literature and an online database Fossilworks [25].

## 3. Results

### 3.1. Phylogenetic Relationships

**Figure 1:**
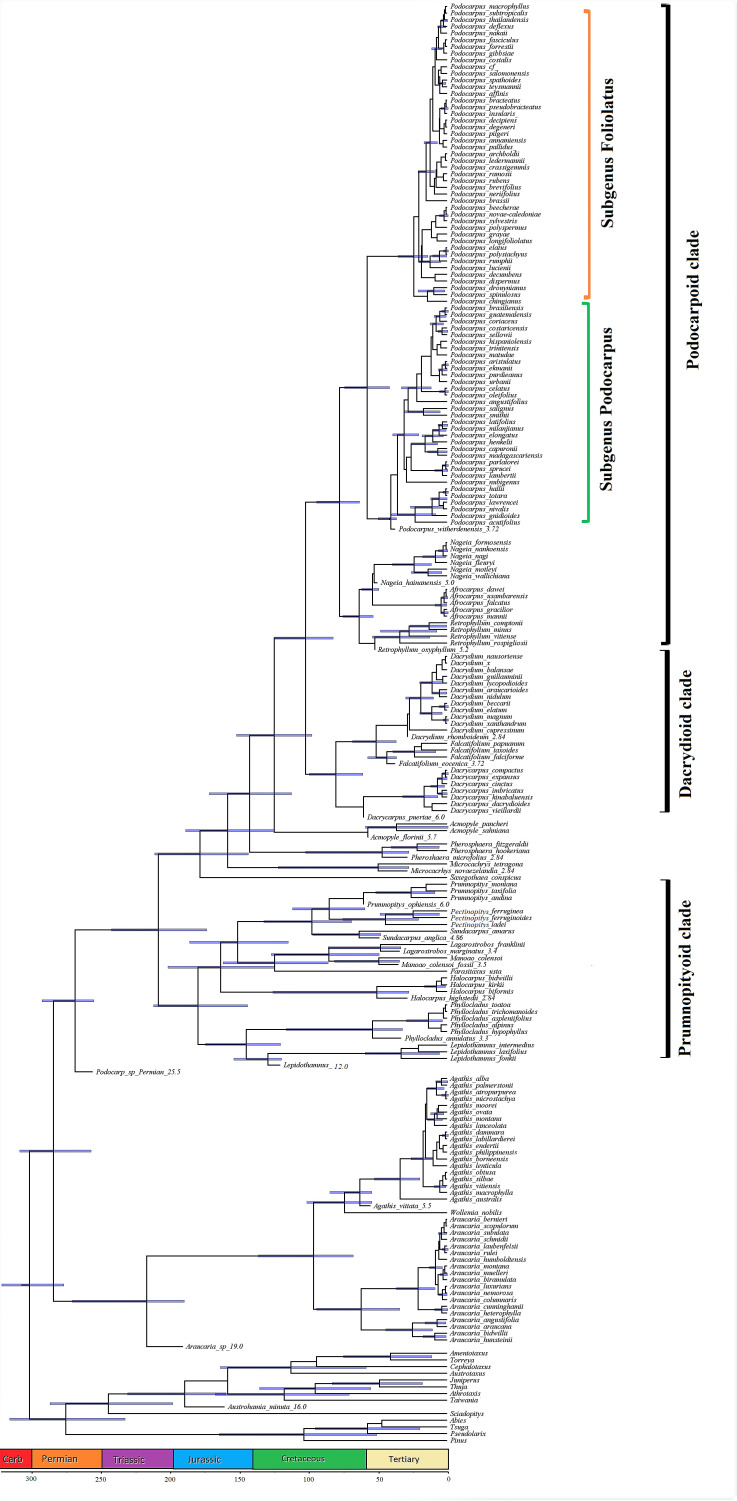
The phylogenetic relationships and divergence time of the 20 extant podocarp genera within Podocarpaceae. Blue node bars indicate the 95% highest posterior density divergence time estimates for the corresponding node. Branch lengths are proportional to time (Ma, millions of years).

The fossil-calibrated phylogenetic tree under the FBD model indicates that the Podocarpaceae and Araucariaceae diverged around the early–mid Permian and the extant podocarp clades split during the mid–late Triassic. The extant podocarp genera have an estimated divergence time of the early Jurassic. The extant podocarp species predominantly show recent diversification from the Oligocene onwards. The phylogeny of the Podocarpaceae shows three major clades, I. Podocarpoid, II. Dacrydioid, III. Prumnopityoid, as well as a distinctive paraphyletic group/grade (Figure 1).

I. Podocarpoid clade—four genera, i.e., *Afrocarpus*, *Nageia*, *Podocarpus* and *Retrophyllum*. The suggested crown age for the Podocarpoid clade is approximately 75 Ma (54–85 Ma). The phylogeny also supports the split of *Podocarpus* into two subgenera i.e., *Foliolatus* and *Podocarpus*.

II. Dacrydioid clade—three genera, i.e., *Dacrydium*, *Dacrycarpus* and *Falcatifolium*. The suggested crown age for the Dacrydioid clade is approximately 75 Ma (54–95 Ma).

III. Prumnopityoid clade—nine genera, i.e., *Lepidothamnus*, *Phyllocladus*, *Manoao*, *Lagarostrobos*, *Parasitaxus*, *Halocarpus*, *Sundacarpus*, *Pectinopitys* and *Prumnopitys*. The newly dated phylogeny shows that the crown age for the Prumnopityoid clade is approximately 175 Ma (150–210 Ma). 

IV. Paraphyletic group/grade—four genera, i.e., *Acmopyle*, *Pherosphaera*, *Microcachrys* and *Saxegothaea*.

### 3.2. Diversity at Genus Level and Distribution

**Figure 2:**
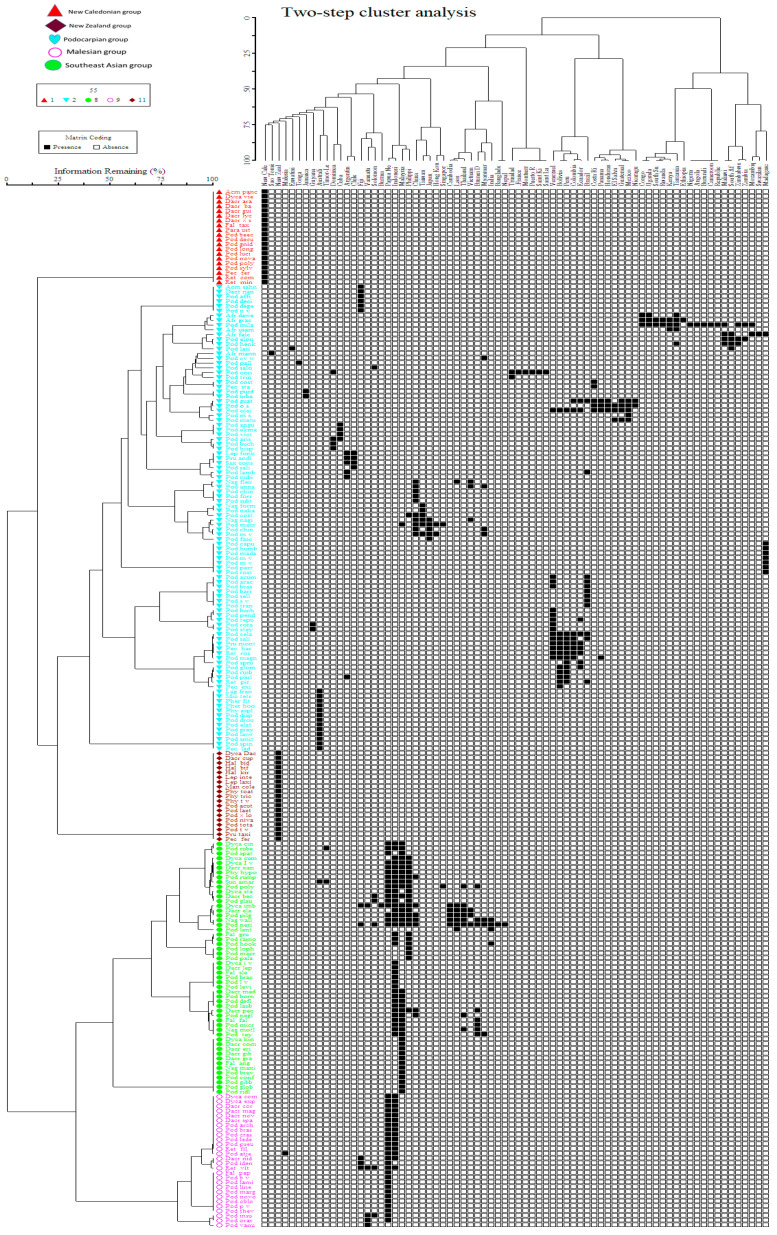
Diagram of two-way cluster analysis. The vertical matrix axis consists of more than 200 podocarp species coded by scientific name and the horizontal axis is the distributed countries (74 countries). The matrix was constructed depending on the presence (black) and absence (white). The species were grouped into five main clusters, I. New Caledonian group, II. New Zealand group and III. Malesian group, IV. Southeast Asian group and V. Podocarpian group.

**Figure 3:**
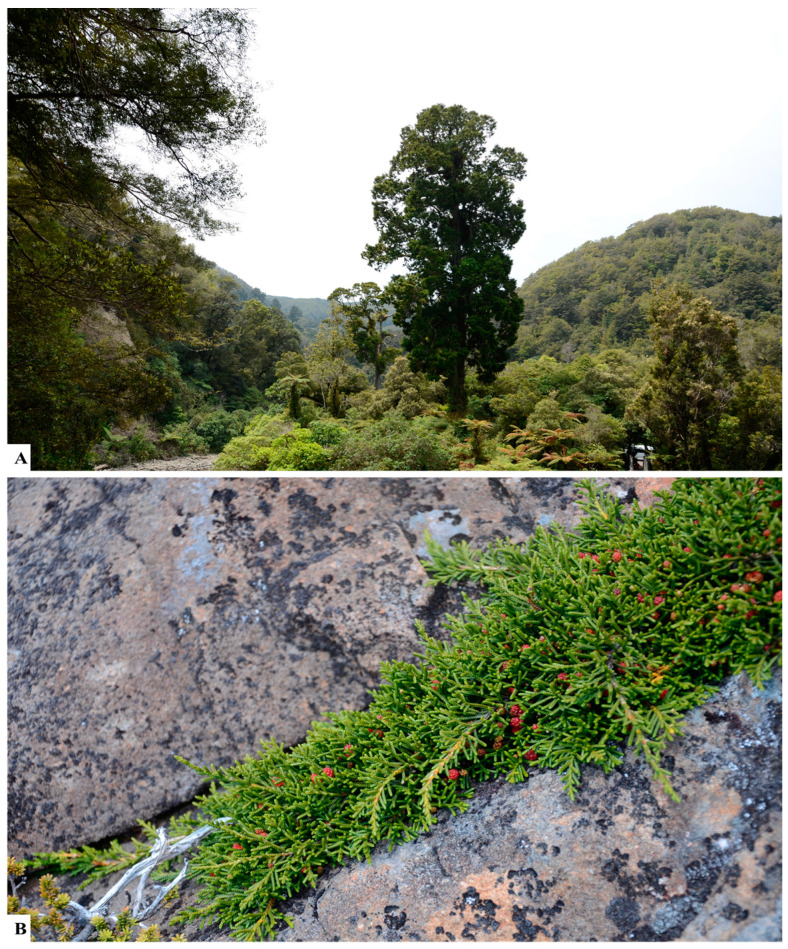
(**A**) *Dacrycarpus dacrydioides* (White pine, Kahikatea) tree in the rainforest of Wellington Kaitoke Regional Park, New Zealand. (**B**) *Microcachrys tetragona* (Strawberry pine) is a creeping shrub in the alpine region of cradle mountain summit, Tasmania.

Currently, in the Podocarpaceae, 20 genera and approximately 219 taxa (201 species, 2 subspecies, 14 varieties and 2 hybrids) are recognized (Table 1). *Podocarpus* is the most speciose genus with approximately 120 species distributed in approximately 70 countries. Two-way cluster analysis of Podocarpaceae species distribution shows five major groups, I. New Caledonian group, II. New Zealand group, III. Malesian group, IV. Southeast Asian group and V. Podocarpian group, widely distributed across several countries (Figure 2). Some of the widely distributed species are *Afrocarpus gracilior* (7 countries) and *A*. *falcatus* (5 countries), *Dacrycarpus imbricatus* (11 countries), *Dacrydium elatum* (7 countries), *Dacrydium pectinatum* (5 countries), *Nageia wallichiana* (11 countries), *Podocarpus coriaceus* (7 countries), *P*. *guatemalensis* (9 countries), *P*. *milanjianus* (15 countries), *P*. *neriifolius* (16 countries), *P*. *oleifolius* (11 countries), *P*. *pilgeri* (9 countries), *P*. *polystachyus* (7 countries) and *Sundacarpus amarus* (6 countries). The current species diversity and distribution is listed in Table 1 and summarized here:

1. *Acmopyle* has two species in New Caledonia (*A*. *pancheri*) and Fiji (*A*. *sahniana*).
2. *Afrocarpus* has five species distributed across Africa.
3. *Dacrycarpus* has nine species and two varieties distributed in New Caledonia, New Zealand, some Pacific Islands and Southeast Asia (Figure 3A).
4. *Dacrydium* has 20 species and two hybrids distributed in New Caledonia, New Zealand, some Pacific Islands and Southeast Asia.
5. *Falcatifolium* has six species distributed in New Caledonia, Papua New Guinea, Indonesia, Malaysia, the Philippines and Brunei Darussalam.
6. *Halocarpus* has three species endemic to New Zealand.
7. *Manoao* has one species distributed in New Zealand.
8. *Pherosphaera* has two species endemic to Australia.
9. *Lagarostrobos* has one species endemic to Australia.
10. *Microcachrys* has one species endemic to Australia (Figure 3B).
11. *Lepidothamnus* has three species distributed in Argentina, Chile and New Zealand.
12. *Nageia* has six species distributed in Southeast Asia to India and Myanmar, Papua New Guinea, the Philippines and Japan.
13. *Parasitaxus* has one parasitic species endemic to New Caledonia.
14. *Phyllocladus* has four species and one variety distributed in Papua New Guinea, Indonesia, Malaysia, the Philippines, New Zealand and Australia.
15. *Podocarpus* is the largest genus, with 120 species, 4 subspecies, 9 varieties and one hybrid and has a wide distribution, occurring in all continents (approximately 70 countries) except Europe and Antarctica.
16. *Prumnopitys* has three species distributed in New Zealand and South America.
17. *Pectinopitys* has six species distributed in New Zealand, Australia, New Caledonia and South America.
18. *Retrophyllum* has six species distributed in New Caledonia, South America and the Pacific Islands.
19. *Saxegothaea* has one species distributed in South America.
20. *Sundacarpus* has one species distributed in Australia, Indonesia, Malaysia, Papua New Guinea (Timor-Leste) and the Philippines.

The Podocarpaceae has its highest diversity of genera in New Zealand (nine) with fewer in other regions New Caledonia and Malesia (eight), Australia (seven), South America (four) and Africa and Asia (two). Of the 20 genera, three are endemic to Australia and two are endemic to New Zealand. Countries with a rich diversity of podocarps include Indonesia (51 species), Papua New Guinea (43 species), Malaysia (39 species), the Philippines (23 species), New Caledonia (20 species), New Zealand (19 species), China (17 species), Venezuela (15 species), Australia (14 species), Brazil, Peru and Bolivia (12 species each), Fiji (11 species), Ecuador (9 species) and Madagascar, Thailand, Brunei Darussalam and Colombia (8 species each). 

### 3.3. Podocarpaceae in Space and Time

This checklist consists of macrofossils assigned to extant podocarp genera and includes more than one hundred taxa from the Cretaceous to the Pleistocene (Table 2). The macrofossils are predominantly recorded from Eocene–Miocene deposits. Australian and New Zealand macrofossil records dominate. Most of the macrofossils are foliage but well-preserved reproductive parts (seed and pollen cones) are also recorded for *Lepidothamnus*, *Lagarostrobos*, *Dacrycarpus*, *Phyllocladus*, *Podocarpus* and *Nageia*. Extant podocarps dominate in the Southern Hemisphere and analysis of extinct taxa assigned to living podocarp genera supports their past importance in the Southern Hemisphere (Table 2). A number of extinct species assigned to Podocarpaceae genera have been reported from Australia, New Zealand and South America [26]. An analysis shows that:

1. *Acmopyle* has eight fossil species recorded (*A*. *antarctica*, *A*. *compactus*, *A*. *engelhardti*, *A*. *florinii*, *A*. *glabra*, *A*. *masonii*, *A*. *setiger* and *A*. *tasmanica*) from the Eocene–Oligocene of the Antarctic Peninsula, Australia, New Zealand and Argentina [27,28,29,30,31,32]. The fossil record shows a wider past distribution of *Acmopyle* compared to its current occurrence in New Caledonia and Fiji.
2. *Dacrycarpus* has approximately 25 fossil taxa reported *(D. acutifolius*, *D. arcuatus*, *D. carpenterii*, *D. chilensis*, *D. crenulatus*, *D. cupressiformis*, *D. dacrydoides*, *D. geminus*, *D. guipingensis*, *D. involutus*, *D. elandensis*, *D. falcatus*, *D. lanceolatus*, *D. latrobensis*, *D. linearis*, *D. linifolius*, *D. microfolius*, *D. mucronatus*, *D. patulus*, *D. praecupressinus*, *D. puertae*, *Dacrycarpus* sp. (four separate records)) from the Eocene–Early Pleistocene of Australia, New Zealand, China, Argentina [28,30,32,33,34,35,36,37,38,39,40,41,42,43,45,71,76]. Although *Dacrycarpus* currently has no living species in Australia or South America, the fossil record shows its extensive distribution in both those landmasses in the past.
3. *Dacrydium* has approximately 14 fossil taxa reported from Australia (*D. aciculare*, *D*. *fimbriatus*, *D*. *mucronatus*, *D*. *sinuosum*, *D. rhomboideum*, *D*. *tasmanicum* and six taxa described to genus level) and two species from New Zealand (*D*. *microphyllum* and *D*. *waimumuensis*) [32,33,38,39,47,48]. These suggest an Australasian origin in the Late Cretaceous [77].
4. *Falcatifolium* has only one fossil extinct species (*F. eocenica*) from the Middle Eocene of Victoria, Australia [49]. Currently, Australia has no living species of *Falcatifolium*.
5. *Nageia* has four extinct species (*N*. *hainanensis*, *N*. *maomingensis*, *N*. *ryosekiensis*, *N*. *sujfunensis*), from the Early Cretaceous–Eocene in China, Southwest Japan, and Far East Russia [53,54,55,56,78]. This demonstrates that *Nageia* had a wider past distribution and occurred in Japan and Russia.
6. *Retrophyllum* has four extinct species (*R*. *australe*, *R*. *oxyphyllum*, *R*. *superstes*, *R*. *vulcanense*) recorded from the Cretaceous–Miocene in Australia, Argentina, and New Zealand [28,41,65,74]. Currently, Australia and New Zealand have no living species of *Retrophyllum*.
7. *Podocarpus* has at least 16 extinct species reported and approximately seven taxa identified at the genus level. These are *Podocarpus platyphyllum*, *P*. *sinuatus*, *P*. *strzeleckianus*, *P*. *tasmanicus* and *P*. *witherdenensis* (Eocene) from Australia; *P*. *travisiae* and *P*. *alwyniae* (Miocene) from New Zealand; *P*. *andiniformis*, *P*. *araucoensis* and *P*. *inopinatus* (Eocene–Miocene) from South America; and *P*. *pliomacrophyllus*, *P*. *yunnanensis* and *P*. *forrestii* (lower Pliocene) from China [27,28,38,42,65,69,70,71,79].
8. *Halocarpus* has a single occurrence of one extinct species (*Halocarpus highstedii*) reported from the Oligocene–Miocene of New Zealand [39].
9. *Manoao colensoi* has a fossil record from the Oligocene of Cethana, Tasmania (Australia) (reported as *Lagarostrobos colensoi*) [38], showing that this current New Zealand endemic genus was once also distributed in Australia.
10. *Lepidothamnus* has three fossil records: *L. intermedius* from the Miocene in New Zealand [30], *L. diemenensis* from the Eocene of Hasties, Tasmania [28] and an undescribed extinct species from the middle Cretaceous of Winton, Queensland [51]. This indicates a wider distribution of *Lepidothamnus* in the Late Mesozoic across the Southern Gondwana regions [51].
11. *Lagarostrobos* has two fossil records, e.g., *L. marginatus* from the Early Oligocene and the extant *L. franklinii* from the Early Pleistocene in Tasmania, Australia [33,34,39,45,50]. Current and macrofossil records suggest a narrow distribution and endemism to Australia for this genus.
12. *Phyllocladus* has seven fossil species described, including records of the extant *P. aspleniifolius* (the fossil species are *P. aberensis*, *P*. *annulatus*, *P*. *elongatus*, *P*. *lobatus*, *P*. *morwellensis*, *P*. *palmeri*) and six at genus level from Late Eocene–Pliocene in Australia, New Zealand and Antarctica (Cretaceous) [38,39,47,50,57,58,59,61,62,63,64]. *Protophyllocladus* Berry [80], an extinct genus that resembles the foliage morphology of *Phyllocladus*, is recorded from the Jurassic and Cretaceous of the United States and southwestern Canada, Western Greenland, Serbia, Romania, Portugal, Kazakhstan, Japan, northeastern Russia, Germany [81]. Dörken et al. [82] considered that it is unlikely that *Protophyllocladus* is closely related to *Phyllocladus*. Wagstaff [83] suggested that extant species are the remnants of one of the recently diverged lineages of *Phyllocladus*, but there is no unequivocal fossil evidence to support this.
13. *Prumnopitys* has two extinct species (*P. portensis* and *P. tasmanica*) and a fossil record of one extant species (*P. montana*) from the Eocene in Australia and one extinct species (*P. opihiensis*) and a fossil record of one extant species (*P. taxifolia*) from the Cretaceous–Miocene in New Zealand [28,30,42,43,72].
14. *Sundacarpus* has two extinct species: *S*. *anglica* and *S*. *tzagajanicus*, from the Eocene–Oligocene in Australia and Cretaceous–Paleocene in Argentina, respectively [75].
15. *Pherosphaera* has two extinct species: *P*. *sommervillae* (*Microstrobos sommervillae*) from the Early Eocene and *P*. *microfolius* (*Microstrobos microfolius*) from the Oligocene–Miocene of Tasmania [31,33].
16. *Microcachrys* has one extinct species (*Microcachrys novae-zelandiae*) from the Oligo-Miocene of New Zealand and fossils of the extant *Microcachrys tetragona* from the Early Pleistocene of western Tasmania [34,45,52].

### 3.4. Chromosomal Number

The chromosomal number in the Podocarpaceae varies from x = 9 to x = 19 (*Acmopyle* x = 10, *Afrocarpus* x = 12, *Dacrycarpus* x = 10, *Dacrydium* x = 10, *Falcatifolium* x = 10, *Halocarpus* x = 9, 11, 12, *Lagarostrobos* x = 15, *Lepidothamnus* x = 14, 15, *Manoao* x = 10, *Microcachrys* x = 15, *Nageia* x = 18, *Parasitaxus* x = 18, *Pectinopitys* x = 19, *Pherosphaera* x = 13, *Phyllocladus* x = 9, *Podocarpus* x = 10, 11, 17, 18, 19, *Prumnopitys* x = 18, *Retrophyllum* x = 10, *Saxegothaea* x = 12 and *Sundacarpus* x = 12) [84]. 

## 4. Discussion

### 4.1. Phylogenetic History of the Podocarpaceae

Molecular studies suggest Araucariaceae as the sister family of Podocarpaceae, although these families are morpho-anatomically divergent [4,11,12,13], which was also supported by our results. Previous molecular and fossil records suggest that podocarps originated in the Triassic–Jurassic in Gondwana [12,85], or the Early Cretaceous [10], or Late Triassic [13], but recent podocarp fossils from Jordan push back the origin of the Podocarpaceae to the Permian (Figure 1) and show that they survived the “great dying” at the end of Permian [86,87]. Our results suggest that *Lepidothamnus* and *Phyllocladus* diverged in the Late Jurassic, when incorporating the oldest *Lepidothamnus* fossil record [51,88], which is earlier than the previous estimate of mid-Cretaceous–early Paleogene [10,12] and Early Jurassic [13]. Our studies recognized the presence of three major Prumnopityoid, Dacrydioid and Podocarpoid clades and a paraphyletic group similar to Chen et al. [13].

Several studies based on both morphological [7,89,90,91,92] and molecular [7,92,93,94,95,96] studies have been published evaluating the phylogenetic relationship among different genera of the Podocarpaceae. Based on morphology and 18S rDNA, Kelch [7] concluded that the Podocarpaceae are monophyletic except for *Podocarpus* (paraphyletic) and *Dacrydium* (polyphyletic). Conran et al. [93], based on molecular analysis (plastid *rbcL*), reported that Podocarpaceae are polyphyletic and supported the separation of *Afrocarpus* from *Podocarpus* and its placement as sister to *Retrophyllum* instead of *Nageia*, and also suggested that *Podocarpus* is monophyletic, a conclusion supported by Sinclair et al. [94]. Biffin et al. [85], based on their molecular studies of 94 Podocarpaceae species, reported that *Podocarpus* is closely related to *Nageia*, *Afrocarpus* and *Retrophyllum*. Knopf et al. [92] investigated the phylogeny of 145 species (including 77 species of *Podocarpus*) of Podocarpaceae based on morphological, anatomical and DNA sequences (*rbcL*, nrITS1 and *NEEDLY*). Their most significant findings were the support of subgenera in *Podocarpus*, the transfer of *Sundacarpus amarus* to *Prumnopitys* and the incorporation of the Phyllocladaceae into the Podocarpaceae as *Phyllocladus*. Lu et al. [11] reported two monophyletic sister groups: the Dacrydioid group (*Dacrycarpus*, *Dacrydium* and *Falcatifolium*) and the Podocarpoid group (*Retrophyllum***–***Nageia* subclade and the *Afrocarpus–Podocarpus* subclade). Little et al. [95] used DNA barcoding (*matK*, *rbcL* and nrITS2 DNA barcodes) for the identification of Podocarpaceae (18 genera and 145 species) and to construct a phylogenetic tree. Quiroga et al. [97], based on molecular and fossil data, reported that *Podocarpus* originated in late Cretaceous–early Paleogene (~63 Ma) and supported the two subgenera in *Podocarpus*. Leslie et al. [96], using more comprehensive sampling and markers, recognized 19 genera and supported the division of *Podocarpus* into two subgenera. Recently, Page [75] again split the genus *Prumnopitys* into three genera (*Prumnopitys*, *Sundacarpus* and the new genus *Pectinopitys*) based on morphological and molecular data. The current phylogeny supports the division of the 20 genera of podocarps into main three clades and a paraphyletic grade (Figure 1). Similarly, the current phylogeny also recognizes and supports the division of *Podocarpus* into two subgenera, i.e., *Podocarpus* and *Foliolatus* [12,13,92,97].

### 4.2. Historical Taxonomic Treatment

The most extensive taxonomic studies on the Podocarpaceae have been by de Laubenfels, Buchholz, Gray and Page, with many other contributions, which are summarized in Table 3.

Initially, podocarps were placed in two genera, *Podocarpus* and *Dacrydium*, mainly based on leaf morphology [98]. Several early taxonomists including Gordon [111] and Philippi [112] recognized variation in *Podocarpus* and *Dacrydium* and classified them into several sections, subgenera, and subgroups. From the 1960s onwards, *Podocarpus* was then divided into eight genera and *Dacrydium* into five. Based on leaf morphology and anatomy, *Podocarpus* was initially divided into eight sections (*Afrocarpus*, *Dacrycarpus*, *Eupodocarpus*, *Microcarpus*, *Nageia*, *Polypodiopsis*, *Stachycarpus* and *Sundacarpus*). After a more detailed examination, de Laubenfels [113] raised section *Dacrycarpus* to the genus *Dacrycarpus*. Quinn [114] suggested raising the eight sections of *Podocarpus* to generic level and de Laubenfels [115] raised the section *Microcarpus* to generic level as *Parasitaxus.* Later, de Laubenfels [104] revised the genus *Podocarpus* into 18 sections and described 94 species. Page [106] raised section *Sundacarpus* into the genus *Sundacarpus*, section *Polypodiopsis* to the genus *Retrophyllum*, section *Nageia* to the genus *Nageia* and section *Afrocarpus* to the genus *Afrocarpus*. Some taxonomists reject these changes of status [116,117,118]. Page [107] divided the Podocarpaceae into 17 genera (*Phyllocladus* was excluded and *Sundacarpus* was included). Biffin et al. [85] recognized three major clades, i.e., the Podocarpoid clade (*Afrocarpus*, *Nageia*, *Podocarpus*, *Retrophyllum*), the Dacrydioid clade (*Dacrydium*, *Dacrycarpus*, *Falcatifolium*) and the Prumnopityoid clade (*Halocarpus*, *Lagarostrobos*, *Manoao*, *Parasitaxus*, *Prumnopitys*). 

The concept of the separate family Phyllocladaceae has been supported in several different studies [93,107,119], and while this is no longer regarded as valid, its status as the genus *Phyllocladus* has been well supported by other taxonomists and recent phylogenetic studies [12,13,83,92,94,95,120,121,122,123,124].

Our studies recognize 20 extant genera (*Acmopyle*, *Afrocarpus*, *Dacrycarpus*, *Dacrydium*, *Falcatifolium*, *Halocarpus*, *Lagarostrobos*, *Lepidothamnus*, *Manoao*, *Microcachrys*, *Nageia*, *Parasitaxus*, *Pectinopitys*, *Pherosphaera*, *Phyllocladus*, *Podocarpus*, *Prumnopitys*, *Retrophyllum*, *Saxegothaea* and *Sundacarpus*), two subgenera in both *Podocarpus* (*Podocarpus* and *Foliolatus*) and *Prumnopitys* (*Prumnopitys* and *Botryopitys*) in Podocarpaceae, similar to Page [75] and Yang et al. [1], Chen et al. [13] proposes the splitting of *Prumnopitys* into *Prumnopitys* and *Pectinopitys* but reported 19 genera for Podocarpaceae. Our checklist enlists 201 living species for Podocarpaceae than previously reported 181 species by Yang et al. [1], which will increase the total number of conifer species from 702 to 722.

### 4.3. Current Diversity and Distribution of Podocarpaceae

Podocarps occur mainly in the Southern Hemisphere, although some genera extend northward, i.e., subtropical China and Japan and to Mexico and the Caribbean [125]. The living species of Podocarpaceae are a small representation of a once highly diverse group [55,126]. Today, several genera have low species representation (e.g., monospecific in *Manoao*, *Lagarostrobos*, *Microcachrys*, *Parasitaxus* and *Saxegothaea* and two in *Acmopyle* and *Pherosphaera*), although the fossil record suggests a more extensive diversity for at least some of these genera in the past. The center of diversity for the Podocarpaceae is Australasia (New Caledonia, Tasmania, New Zealand and Malesia), South America (Andes mountains), Indo-China and the Philippines [125,127].

**Figure 4:**
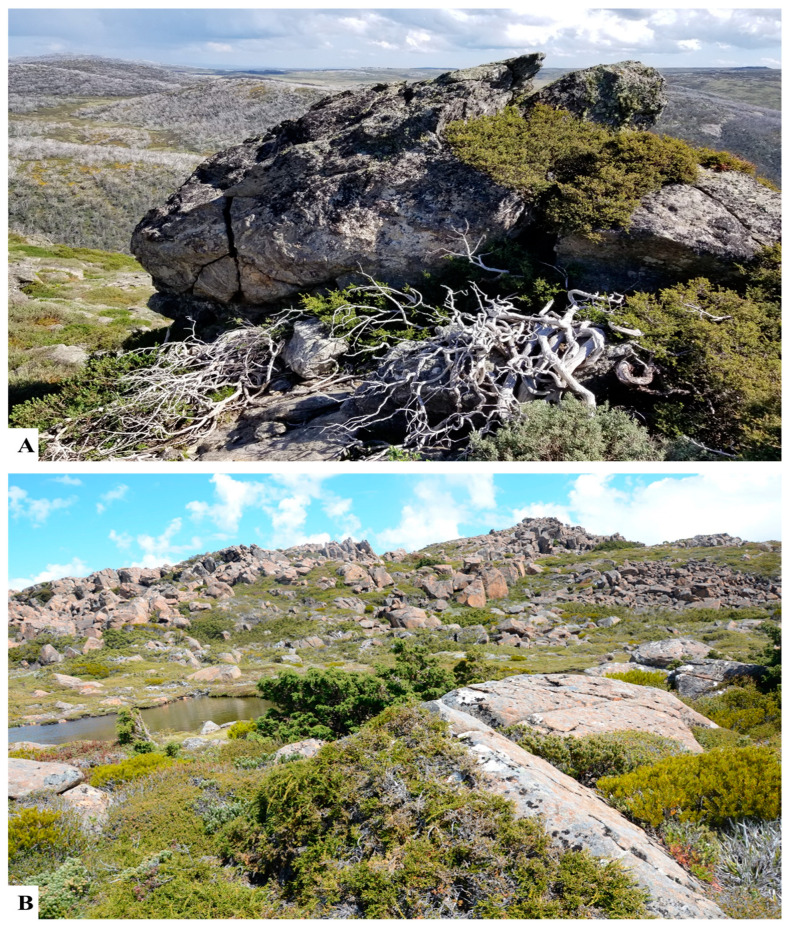
(**A**) *Podocarpus lawrencei* in the alpine region, Mount McKay Falls Creek, Australia. (**B**) *Pherosphaera hookeriana* and *Microcachrys tetragona* populations in the alpine region of Cradle Mountain summit, Tasmania.

Podocarps favor mostly cool and wet climates but usually do not tolerate extreme cold like some Northern Hemisphere conifers [128]. However, some temperate Podocarpaceae species occur as shrubs and prostrate woody plants above the tree line in the alpine ecosystems of Tasmania, Victoria, and New Zealand (Figure 4).

The tropical podocarps are mostly confined to mountain forests and heathlands and nutrient-poor habitats in the lowlands, although some also grow in forest understories. Temperate podocarps are good competitors in nutrient-poor soils probably because the light is more easily available within the incomplete canopies, but they are outcompeted in nutrient-rich soil as the canopy and forest floor is occupied by angiosperms and the growth of new individuals is slow due to shading. Such conditions favor broad-leaved podocarps (*Nageia* and broad-leaved *Podocarpus* species are shade-tolerant) and exclude imbricate-leaved podocarps due to competition [128]. This is supported by Adie and Lawes [129], who concluded that African podocarps are not lowland rainforest survivors but are temperate forest relicts.

### 4.4. Historical Biogeography

The historical reconstruction of Podocarpaceae confirms that it is a Southern Hemisphere family that was initially centered in Gondwana [130]. Leslie et al. [12] suggest that Podocarpaceae diversified in the Cretaceous and earliest Cenozoic after its appearance in the Triassic of Gondwana. Klaus and Matzke [10], based on the reconstruction of ancestral ranges, suggested that podocarps originated in the Early Jurassic in what is today Central–South America, Australia, and New Zealand. The family subsequently dominated Australasia and Southern America and later (and through to the present) in Malesia [77]. However, the discovery of macrofossils of podocarps from the early Permian of Jordan [86,87] will require a re-assessment of the early history of the family [88].

Klaus and Matzke [10] used living taxa to reconstruct the ancestral ranges and suggested that the ancestral area for the Podocarpoid clade is the Australia–New Guinea–Malesian region; for the Dacrydioid clade it is New Caledonia; for the Prumnopityoid clade it is New Zealand and for the paraphyletic group/grade, South America to Australia. Macrofossil evidence and the historical biogeographic reconstruction by Klaus and Matzke [10] support an Australian origin of *Podocarpus* and multiple dispersals to South America, Asia, New Zealand, Malesia, and New Caledonia. Morley [77] concluded that *Podocarpus* dispersed into South Asia in the Late Eocene, either by dispersal from India or by multiple long-distance dispersal events from Australia. Similarly, he concluded that *Podocarpus* was possibly present in Africa during the mid-Cenozoic but its dispersal to West Africa occurred by island-hopping in the late Pliocene. According to Nieto-Blázquez et al. [131], *Podocarpus* species in the Caribbean are the result of colonization from the Andes during the Eocene to Oligocene (c. 45–31 Ma). Fossil records and living species distributions of *Nageia* support an Asian origin [10,54]. The living species of *Afrocarpus* strongly support an African origin for that genus. The living taxa and fossil record suggest a Gondwanan origin of *Retrophyllum*, with it evolving by the early Eocene [65,74]. Although the historical biogeographic reconstruction produced by Klaus and Matzke [10] suggests the origin of *Dacrydium* in New Caledonia, the macrofossil record strongly suggests an Australasian origin [32,39]. Morley [77] also concluded that *Dacrydium* originated in Australasia in the Late Cretaceous and dispersed to Southeast Asia in the Early Oligocene, probably by island-hopping (e.g., it dispersed to the Ninety East Ridge by the Paleocene and to India by the Early Eocene and later expanded to Japan during the Middle Miocene climatic optimum). According to Wu et al. [36,76], *Dacrycarpus* also had an Australasian origin during the Late Cretaceous. *Dacrycarpus* was present by the Eocene in Patagonia, supporting biogeographic connections during the warm Eocene from Patagonia to Australasia across Antarctica [35]. According to Morley [77], *Dacrycarpus* dispersed to New Guinea from Australia by the late Miocene and then during the mid-Pliocene, it island-hopped to Borneo, and during the Pleistocene, to Sumatra and the Malay Peninsula. However, the *Dacrycarpus* megafossil from the Miocene of South China shows its earlier arrival to Asia from the Southern Hemisphere and China during Late Miocene [36]. Paleoclimatic studies also support the existence of *Dacrycarpus* in high-precipitation areas and explain its possible extinction in Australia as that continent dried [36,85]. *Dacrycarpus* possibly became extinct around the Paleogene–Neogene transition from both South America and Antarctica and during the Neogene from Australia [36]. Klaus and Matzke’s [10] historical biogeographic reconstruction suggests that *Falcatifolium* originated in the Fiji–New Guinea region around the Late Eocene. However, the fossil record of *Falcatifolium* from the Middle Eocene of Australia suggests an Australian origin [49], *Falcatifolium* probably dispersed later to New Caledonia and Papua New Guinea [84].

Klaus and Matzke [10] also concluded that the Prumnopityoid clade originated in New Zealand around the mid-Cretaceous. However, a recent phylogeny of the podocarps suggests an Early to Mid-Jurassic origin for this clade (Figure 1). Leslie et al. [5] and Wang and Ran [84] reported that the phylogenetic divergence of Podocarpaceae shows that the three genera (*Lepidothamnus*, *Podocarpus* and *Prumnopitys*) were dispersed from Australia to South America through Antarctica. A *Lepidothamnus* macrofossil from the middle Cretaceous of Winton, Queensland [51,88] also supports its Australian origin. The living and macrofossil records of *Phyllocladus* indicates a Gondwanan origin and wider distribution. *Phyllocladus* dispersed to New Guinea by the late Miocene and then, during the mid-Pliocene, it island-hopped to Borneo [77]. The extant and extinct species (*Halocarpus highstedii* from the Oligocene–Miocene) are endemic to New Zealand [39]. Today, *Manoao* is a monotypic endemic genus to New Zealand but one fossil specimen from the Oligocene (35 Ma) of Cethana, Tasmania (Australia) is similar to that of *Manoao colensoi* (reported as *Lagarostrobos colensoi*), showing this genus was once present in Australia [38]. *Parasitaxus* is a monotypic endemic genus to New Caledonia with no macrofossil records. *Lagarostrobos* is also a monotypic endemic genus in Tasmania and the macrofossil records from Early Oligocene to Early Pleistocene are also restricted to Tasmania [33,34].

*Prumnopitys* has three living species distributed in New Zealand and South America. The macrofossil records (Cretaceous–Miocene) demonstrate a Gondwanan origin and wider distribution [43,75]. Although *Sundacarpus* is now a monotypic genus, the macrofossil records (*S. anglica* from England and *S. tzagajanicus* from Russia) from the Uppermost Cretaceous and Eocene show a wider past distribution [75]. *Pectinopitys* is widely distributed in New Zealand, Australia, New Caledonia, and South America, but with no macrofossil record.

Klaus and Matzke [10] conclude that *Acmopyle* originated in New Caledonia, but macrofossils from the Eocene–Oligocene suggest a Gondwanan origin [27,28,29,30,31,32]. *Microcachrys* is now endemic to Australia but is also present in the Oligocene–Miocene of New Zealand [52]. *Saxegothaea* is the oldest genus in the family and is part of an ancient lineage endemic to South America. *Pherosphaera* has two living species and two macrofossils from Australia [33].

### 4.5. Eco-Physiological Adaptations

Most podocarps have evolved flattened leaves and fleshy seed cones, which enable them to survive in low-light conditions beneath the tree canopy and disperse their seeds biotically [85,88,132,133]. Podocarps mature as trees or shrubs. Some of the most significant ecophysiological adaptations and strategies are discussed here.

#### 4.5.1. Seed Cone Morpho-Anatomy

The Podocarpaceae have evolved distinct seed cone morphotypes and display marked variation in functional traits across the 20 genera [88,133,134]. Most podocarp genera produce fleshy seed cones utilizing the epimatium, aril, bracts, receptaculum or a combination of these [109]. *Podocarpus* is the largest genus in the Podocarpaceae and has a cone composed of one or two seeds covered mostly by a papery and sometimes a fleshy epimatium [10,109]. Several podocarp genera have cones with a brightly colored, fleshy receptaculum [10,88,134].

#### 4.5.2. Leaf Morpho-Anatomy

The Podocarpaceae is prominent in many mixed conifer/broadleaf vegetation types in the Southern Hemisphere, and they exhibit great variation in leaf morphology across the 20 genera [135]. The diversity in leaf morphology of Podocarpaceae is remarkable, ranging from uni-veined needle and scale-like leaves to multi-veined broad leaves. Podocarpaceae foliage can be divided into two main types, imbricate (*Dacrycarpus*, *Dacrydium*, *Halocarpus*, *Manoao*, *Lagarostrobos*, *Lepidothamnus*, *Microcachrys*, *Pherosphaera* and *Parasitaxus*) and broad (flattened) leaved (*Acmopyle*, *Nageia*, *Afrocarpus*, *Falcatifolium*, *Phyllocladus*, *Podocarpus*, *Retrophyllum*, *Pectinopitys*, *Sundacarpus*, *Prumnopitys* and *Saxegothaea*). These genera have leaves either spirally arranged or in opposite pairs. Most Podocarpaceae species possess flattened or composite leaves (in 11 genera and more than 140 species) and this may be an adaptation to light requirements, as most of these species grow in the understory of forests with a low-light environment and are unable to reach the canopy level and high sunlight [9] unless a canopy gap occurs. *Nageia* is characterized by having leaves with multiple parallel veins [55]. All *Phyllocladus* species have evolved multi-veined phylloclades (Supplementary Figure S1), probably to compete with angiosperms for light [9,82,136]. *Acmopyle*, *Dacrycarpus* and *Falcatifolium* have bilaterally flattened leaves, lacking a true petiole. Leaf dimorphism is present in many genera of Podocarpaceae (Supplementary Figure S2). All other broad-leaved species have bifacially flattened broad leaves [135].

#### 4.5.3. Pollen Morphology

All conifer species are wind-pollinated and those in the Podocarpaceae (except *Saxegothaea*) and Pinaceae have developed special wing-like structures called sacci [2]. The Podocarpaceae usually have saccate pollen with a tectate exine but usually with a smaller grain than the Pinaceae [137]. Pollen of all genera are bi-saccate except *Microcachrys*, *Pherosphaera* and *Dacrycarpus*, which are tri-saccate, and *Saxegothaea* which does not have sacci [91,138,139]. Because of this, Erdtman [138] suggested shifting *Saxegothaea* to the Araucariaceae, while Gaussen [103] and Woltz [140] suggested promoting it to the new family Saxegothaeaceae. The fossil pollen record of the Podocarpaceae is not considered here but is in need of revision, with much important data currently difficult to assess without expert comment on the validity of published interpretations.

### 4.6. Dispersal Biology

The Podocarpaceae are predominantly zoochorous as their main seed dispersal mechanism, although some genera have other dispersal strategies [141]. The zoochorous mode of dispersal is reported in *Dacrycarpus*, *Halocarpus*, *Dacrydium*, *Microcachrys*, *Afrocarpus*, *Nageia*¸ *Podocarpus*, *Lepidothamnus*, *Phyllocladus*, *Parasitaxus*, *Manoao*, *Sundacarpus*, *Falcatifolium*, *Retrophyllum*, *Prumnopitys* and *Pectinopitys* [88,134]. Klaus and Matzke [10] reported that 11 genera of Podocarpaceae show endozoochory, two (*Prumnopitys* and *Afrocarpus*) epizoochory and seven genera are not ornithochorous. Barochory is present in *Pherosphaera* and *Saxegothaea*. Hydrochory and zoochory are reported in *Retrophyllum comptonii*, *R*. *minor* and *Lagarostrobos* [109].

Leslie et al. [96] reported that cone morphology and seed size are co-evolved in a correlated pattern in animal-dispersed conifers and animal-dispersed species have a relatively larger seed size to attract animals. Similarly, climate change (higher temperatures or water stress in drier conditions) can affect the evolution of cone shape. Interpreting the cone morphology and animal dispersal in Podocarpaceae is difficult because animal-dispersed seeds (fleshy cones) evolved many times in the deep past (from the Cretaceous or even earlier, based on ancestral reconstruction) [88,96,134]. *Podocarpus* can be interpreted as zoochorous and mainly bird-dispersed due to their colorful fleshy receptacle and epimatium. Bird and bat dispersal have been reported from South African podocarps [142]. The Emu (*Dromaius novaehollandiae*) is a large bird with a wide distribution range in Australia and it is the main disperser of *Podocarpus drouynianus* in southwestern Australia, keeping the seeds for up to 50 h in the digestive tract and dispersing them several kilometers [143].

### 4.7. Ecology of Podocarpaceae

The major Southern Hemisphere conifer family Podocarpaceae is different in morphology, functional physiology, and ecology from the Northern Hemisphere’s major conifer family Pinaceae. Pinaceae are successful in Northern Hemisphere forests, where angiosperms are outcompeted during freezing temperatures, and also occur in low-rainfall areas. Podocarp species are more abundant and compete more successfully with broadleaf angiosperms in the tropical montane forest through multiple morphological and anatomical adaptations but in most cases avoid low-rainfall areas [144]. Ecologically, podocarps have a highly conserved association with the conifer families Araucariaceae and Cupressaceae and with the angiosperm families Nothofagaceae, Winteraceae and Cunoniaceae [9,136]. However, ecological data are lacking for most of the species in these families [4]. 

Podocarps are unable to bear extreme cold temperate but can tolerate moderate frosts [128] and some exist as alpine shrubs in relatively cold climates (e.g., alpine Tasmania) where permanent snow is uncommon (Figure 4). They possess broad to scale leaves, phylloclades and fleshy cones and they are adapted to a range of conditions from alpine to lowland, understory environments beneath a dense canopy, semi-aquatic (*Retrophyllum minus*), drought-and fire-prone conditions (*Podocarpus drouynianus*). The only parasitic gymnosperm (*Parasitaxus usta*) grows on the roots of another podocarp species (*Falcatifolium taxoides*). The occurrence of extant species of Podocarpaceae in angiosperm-dominated humid forests is of great interest to ecologists and paleontologists. The Podocarpaceae have preferred wet climates throughout their history [77] and nutrients are a stronger limiting factor for their distribution than the temperature [145], with Coomes and Bellingham [128] reporting that within temperate and tropical rainforests with few exceptions, podocarps are well adapted to nutrient-poor soils.

Coomes and Bellingham [128] evaluated the ecological similarities and differences of temperate and tropical podocarps. They concluded that angiosperm diversification and expansion during the Late Cretaceous was responsible for driving conifers from the lowland tropics and mesic temperate regions due to inferior reproductive competitiveness. However, Bond [146] and Midgley and Bond [147] challenged this view and hypothesized that the physiological traits of conifers (slow seedling establishment and later growth) put them at a disadvantage in competitive regeneration in changing climates (increasing cold and droughts) and habitats (nutrient-poor soil, poorly drained soil, and low light). Podocarps are predominantly slow-growing with low photosynthetic capacity per unit leaf mass and per unit leaf area compared with angiosperms with the same leaf are [128]. The studies that evaluated the growth of podocarps in different habitats lead to the conclusion that podocarp growth is slow compared to other conifers and to angiosperms (e.g., in lowland cool temperate forest, the growth rate is half that of angiosperms [148], and in subalpine shrublands, podocarps grow more slowly than several angiosperm species [149]. In the nutrient-rich soil of southern New Zealand, even tree ferns grow faster than podocarps [150,151]. 

Brodribb [144] argues that drought is one of the major agents that prevents podocarp success at high altitudes in the Southern Hemisphere. The Late Cenozoic was a major drying period in the temperate region and resulted in the contraction and extinction of Australian and other southern podocarps [152]. The cool and wet conditions (on the continental margins of Gondwana) necessary for the diversification of the Podocarpaceae favor the theory of the drought sensitivity of Podocarps [135,153]. High wood density (that lowers hydraulic efficiency) and leaf sclereids (that collapse under water tension, which results in a loss of hydraulic and photosynthetic function in the leaf) are also present in the broad-leaved tropical podocarps and may be the cause of poorer drought performance and weak competition in drier forests but favor cool, shady, and wet regions of the Southern Hemisphere for podocarp persistence [135,144,154]. In contrast, the Pinaceae have tough and waxy needle-like leaves, lower wood density, fewer sclereids and a high photosynthetic rate, making them resistant and adaptable to drought and freezing temperatures that are common in parts of the Northern Hemisphere [144,155]. This also provides a possible insight into why podocarps are today almost absent from the Northern Hemisphere, despite their potential for long-distance dispersal. A few podocarps are tolerant of drier regions, e.g., *Afrocarpus falcatus* (southern Africa), *Podocarpus drouynianus* (Western Australia) and *Halocarpus bidwillii*, *Phyllocladus alpinus* and *Podocarpus laetus* (dry lowland forests of New Zealand) [134]. Podocarp morphology is unusual compared to other conifers, since, despite possessing thick tracheid walls that are vulnerable to embolism at low tensions [154]. (Pittermann et al., 2006b), they also have high hydraulic resistance across pit membranes [156] and that makes the implosion of sclereids in podocarp leaves under tension a real possibility [157].

### 4.8. IUCN Conservation Status and Threats

**Figure 5:**
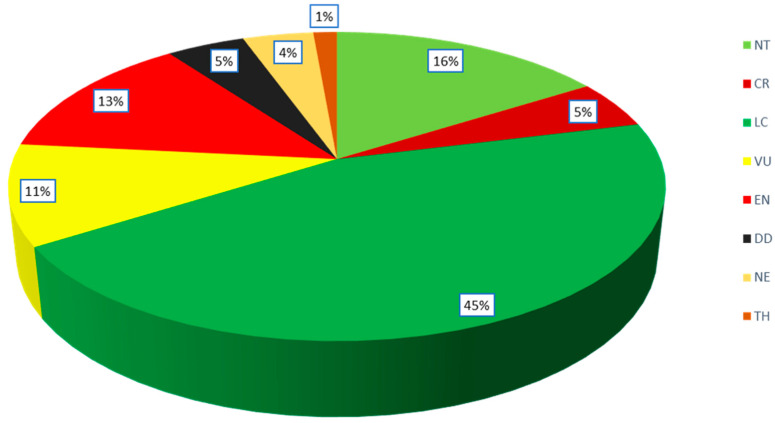
The proportion of current IUCN conservation status of Podocarpaceae species. The conservation status is evaluated according to IUCN Red List categories and criteria, version 3.1 (IUCN Council, Geneva, Switzerland).

The analysis of the available data on the IUCN conservation status of Podocarpaceae shows that 8 species (1 variety) are Critically Endangered (CR), 27 species (2 varieties) are Endangered (EN), 23 species (one subspecies) are Vulnerable (VU), 3 species are Threatened (TH), 33 species (2 varieties) are Near Threatened (NT), 89 species (8 varieties and one subspecies) are Least Concern (LC), 10 species are Data Deficient (DD) and 7 species (2 hybrids) are Not Evaluated (NE) for IUCN status (Figure 5). The Critically Endangered (CR) species are *Acmopyle sahniana* (Fiji), *Pherosphaera fitzgeraldii* (Australia), *Dacrydium guillauminii* (New Caledonia), *Podocarpus urbanii* (Jamaica), *P. costaricensis* (Costa Rica and Panama), *P. decumbens* (New Caledonia), *P*. *palawanensis* (the Philippines), *P. perrieri* (Madagascar) and *P. sellowii* var. *angustifolius* (Brazil). The IUCN conservation status for tropical podocarps states that 5 species are considered critically endangered, 18 species are endangered, and 16 species are vulnerable (Cernusak et al., 2011). The New Caledonian podocarp species are facing serious conservation threats due to their restricted populations (Enright and Jaffré, 2011); i.e., *Retrophyllum minus* (endangered), *Podocarpus decumbens* (critically endangered) *P*. *longefolaliatus* (endangered), *Dacrydium guillauminii* (critically endangered), *Acmopyle pancheri* (nearly threatened) and *Parasitaxus usta* (vulnerable).

**Figure 6:**
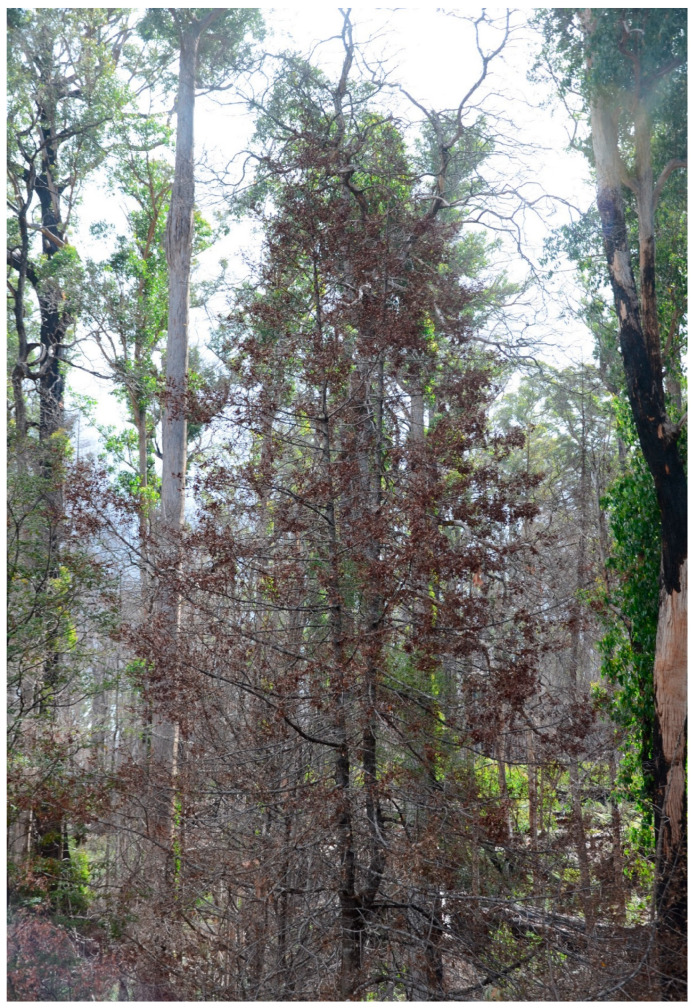
A wildfire in 2020 burnt the Tahune rainforest, Tasmania. This photo is of a burnt Celery-top Pine (*Phyllocladus aspleniifolius*) tree.

Deforestation associated with mining, expansion of tropical agricultural activities and other anthropogenic activities poses a serious threat to tropical podocarps [158]. Deforestation and climate change are also posing a serious threat to montane endemic podocarps [159]. Similarly, more extreme dry seasons are also damaging for tropical podocarps because they are drought and fire intolerant [158]. Wildfire is posing a huge threat to Australian podocarps (Figure 6) and in some areas, the podocarp population has been driven to extinction by these fires [160]. The harvest of podocarp timber has been an important industry, but their slow growth makes it detrimental and unsustainable for the species involved [161]. Mill [162] reported habitat loss, climate change and deforestation as major threats causing the extinction of *Podocarpus* species. Failure of regeneration and aging of the current populations are two major threats for at least some podocarp species [128,163,164]. 

### 4.9. Current Gaps and Future Perspectives

Some clear gaps still exist that need to be filled in order for us to gain a better understanding of the Podocarpaceae and include some of the following aspects:

1. Descriptions and taxonomic treatments of several species from less explored/remote areas such as Papua New Guinea, Malaysia, Indonesia, and New Caledonia are based only on collections of one or a few specimens. Additionally, some of these areas are not well explored and may contain undescribed species.
2. Field-and laboratory-based studies on pollination biology, the reproductive cycle and anatomical structures are not well developed for most podocarps and require further detailed evaluation.
3. Extensive research is required to understand why Podocarpaceae have such remarkable seed cone and leaf morphology.
4. Very few studies report the dispersal biology of podocarp seeds and comprehensive assessments are required to understand the dispersal biology and ecology of podocarps.
5. Despite the several high-quality publications on the leaf cuticle morphology of various genera, a good quality publication is necessary that describes the taxonomic and phylogenetic authenticity of these foliar cuticular diagnostic characters. Similarly, studies are required to assess the infraspecific variation in the leaf morphology for different populations.
6. Phylogenomic and population-based studies are available only for a few *Podocarpus* species (*P*. *matudae*, *P*. *nubigenus*, *P*. *parlatorei*, *P*. *salignus*, *P*. *latifolius*, *P*. *guatemalensis* and *P*. *oleifolius*), and with fairly limited geographic scope (the Americas). With the availability of modern NGS techniques and bioinformatic tools, more comprehensive studies are required to unveil their phylogeny, historical biogeography, speciation, and population history.
7. Only a few studies are available on the historical biogeography of Podocarpaceae and the discovery of new podocarp fossils from the Early Permian (Paleozoic) of Jordan [86,87] questions the Gondwanan origin of the Podocarpaceae. The inclusion of well-placed podocarp fossils will help in better understanding the reconstruction of historical biogeography.
8. Comparative studies of the three Southern Hemisphere conifer families (Araucariaceae, Cupressaceae and Podocarpaceae) to evaluate the impact of these families on the habitats they occupy and their relationships with the rest of the Southern Hemisphere biota.
9. Evolution of photosynthetic units in these three families in response to the closed forests that predated the rise to dominance of the angiosperms and angiosperm-dominated rainforests and then the major aridification of the Southern Hemisphere.
10. A better understanding of the response of podocarp foliage to drought stress and the adaptations that have evolved to deal with the constraint of most podocarps in having only a single vein per leaf is required to better understand the distribution and ecology of the family.
11. Use of species distribution modelling to predict the possible ecological niche and the effect of climate change on species range dynamic.
12. A better understanding of the evolutionary history and biology, ecology and life history are important in conservation efforts, given that so many species are threatened.

## 5. Conclusions

The current study provides a comprehensive overview on the systematics, diversity, hotspots, evolutionary adaptations, and conservation status of podocarps. Podocarps are morphologically more diverse compared to other conifer families and the updated phylogeny based on more extensive macrofossil records broadens our understanding of the evolutionary history of Podocarpaceae. Most podocarp genera currently exhibit low species richness and high endemism and often have disjunct distributions. Today, the Malesian region is the diversity hotspot for living podocarp taxa. However, the fossil record demonstrates wider distributions in the past. *Podocarpus*, *Dacrydium* and *Dacrycarpus* are the most dominant genera (approximately 75% of living podocarps) and have acquired particular morpho-anatomical adaptations that help them to survive in tropical forests. Podocarps demonstrate a remarkable seed cone and leaf diversity compared with other conifers. The genera with fleshy seed cones predominantly rely on bird dispersal. Podocarps are facing serious threats from deforestation, climate change, drought and wildfire, and the need for further targeted research is urgent. Among the conifers, podocarps are less well known and receive less attention than their counterparts that dominate the Northern Hemisphere, despite their remarkable morphological diversity and long evolutionary history. 

## Figures and Tables

**Table 1:** An updated checklist of living podocarp taxa.

S#	Name	Synonyms	Common Name	Distribution in World	IUCN Status
1	*Acmopyle pancheri* (Brongn. and Gris) Pilg.	*Acmopyle alba*, *Dacrydium pancheri*, *Nageia pancheri*, *Podocarpus pectinatus*	New Caledonian acmopyle, Pancher’s acmopyle	New Caledonia	NT
2	*Acmopyle sahniana* Buchholz and N.E. Gray	*Parasitaxus vodonaivalui*	Fijian acmopyle	Fiji (Namosi and near Mt Tomanivi.)	CR
3	*Afrocarpus dawei* (Stapf) C.N.Page	*Afrocarpus mannii subsp. dawei*, *Nageia mannii var. dawei*, *Podocarpus dawei*, *Podocarpus usambarensis var. dawei*	-	Congo, Tanzania (Kagara and Mara provinces), Uganda	NT
4	*Afrocarpus falcatus* (Thunb.) C.N.Page	*Afrocarpus falcatus subsp. gaussenii*, *Afrocarpus gaussenii*, *Decussocarpus falcatus*, *Nageia falcata*, *Nageia falcata var. gaussenii*, *Nageia meyeriana*, *Podocarpus falcatus*, *Podocarpus falcatus*, *Podocarpus gaussenii*, *Podocarpus gracillimus*, *Podocarpus meyerianus*, *Taxus falcata*	Outeniqua yellowwood, Bastard Yellow wood	Malawi, Mozambique, South Africa (Eastern Cape Province, KwaZulu-Natal, Limpopo Province, Mpumalanga, Western Cape), Swaziland, Madagascar	LC
5	*Afrocarpus gracilior* (Pilg.) C.N.Page	*Afrocarpus falcatus subsp. gracilior*, *Decussocarpus gracilior*, *Nageia falcata var. gracilior*, *Podocarpus gracilior*	East African Yellow wood	Ethiopia, Kenya, Tanzania, Congo, Rwanda, South Sudan, Uganda	LC
6	*Afrocarpus mannii* (Hook.f.) C.N.Page	*Decussocarpus mannii*, *Nageia mannii*, *Podocarpus mannii*	-	Sao Tomé and Principe	VU
7	*Afrocarpus usambarensis* (Pilg.) C.N.Page	*Afrocarpus mannii subsp. usambarensis*, *Nageia mannii var. usambarensis*, *Podocarpus usambarensis*	African Yellowwood	Tanzania, Kenya (Kyulu Hills, Taita Taveta District)	EN
8	*Dacrycarpus cinctus* (Pilg.) de Laub.	*Bracteocarpus cinctus*, *Bracteocarpus dacrydiifolius*, *Dacrycarpus dacrydiifolius*, *Podocarpus cinctus*, *Podocarpus dacrydiifolius*	-	Indonesia (Maluku, Papua, Sulawesi), Malaysia (Sarawak), Papua New Guinea	LC
9	*Dacrycarpus compactus* (Wasscher) de Laub.	*Bracteocarpus compactus*, *Podocarpus compactus*	Binban Kadzinam, Kaibigl, Kaipik, Pau, Pawa, Uba, Umba, Umbwa	Indonesia and Papua New Guinea	LC
10	*Dacrycarpus cumingii* (Parl.) de Laub.	*Bracteocarpus cumingii*, *Nageia cumingii*, *Podocarpus cumingii*, *Podocarpus imbricata* var. *cumingii*, *Podocarpus imbricatus* var. *cumingii*	-	Indonesia (Sumatera), Malaysia (Sarawak), Philippines	LC
11	*Dacrycarpus dacrydioides* (A.Rich.) de Laub.	*Dacrydium ferrugineum*, *Nageia dacrydioides*, *Nageia excesla*, *Podocarpus dacrydioides*, *Podocarpus thujoides*	Kahikatea (Maori), White Pine	New Zealand	LC
12	*Dacrycarpus expansus* de Laub.	*Bracteocarpus expansus*	-	Indonesia (Papua), Papua New Guinea	LC
13	*Dacrycarpus imbricatus* (Blume) de Laub.	*Bracteocarpus imbricatus*, *Bracteocarpus kawaii*, *Dacrycarpus imbricatus* var. *imbricatus*, *Dacrycarpus imbricatus* var. *patulus*, *Nageia cupressina*, *Podocarpus cupressinus*, *Podocarpus imbricatus*, *Podocarpus javanicus*, *Podocarpus kawaii*, *Thuja javanica*	-	Cambodia, China (Guangxi, Hainan, Yunnan), Fiji, Indonesia (Jawa, Lesser Sunda Is., Papua, Sulawesi, Sumatera), Lao People’s Democratic Republic, Malaysia, Papua New Guinea (Bismarck Archipelago), Philippines, Thailand, Vanuatu, Vietnam	LC
14	*Dacrycarpus imbricatus* var. *curvulus* (Miq.) de Laub.	*Podocarpus cupressina* var. *curvula*, *Podocarpus imbricatus* var. *curvula*	Tjamarah	Indonesia (Sumatra and Java)	LC
15	*Dacrycarpus imbricatus* var. *robustus* de Laub.	*Podocarpus papuanus*, *Podocarpus javanica*, *Podocarpus cupressina*, *Dacrycarpus papuana*	Pierur, tupi, daru and umba	Papua New Guinea, Indonesia (Moluccas), Malaysia (Sarawak) and Philippines (Luzon, Mindanao)	LC
16	*Dacrycarpus kinabaluensis* (Wasscher) de Laub.	*Bracteocarpus kinabaluensis*, *Podocarpus imbricatus* var. *kinabaluensis*	-	Endemic to Mt. Kinabalu in Sabah (Borneo), Malaysia	LC
17	*Dacrycarpus steupii* (Wasscher) de Laub.	*Bracteocarpus steupii*, *Podocarpus steupii*	-	Indonesia (Kalimantan, Papua, Sulawesi), Papua New Guinea, Philippines	NT
18	*Dacrycarpus vieillardii* (Parl.) de Laub.	*Dacrydium elatum* var. *compactum*, *Dacrydium elatum* var. *tenuifolium*, *Nageia tenuifolia*, *Nageia vieillardii*, *Podocarpus taxodioides* var. *tenuifolius*, *Podocarpus tenuifolius*, *Podocarpus vieillardii*	-	New Caledonia	LC
19	*Dacrydium araucarioides* Brongn. and Gris	*Athrotaxis araucarioides*, *Dacrydium arthrotaxoides*, *Metadacrydium araucarioides*, *Podocarpus araucarioides*	-	New Caledonia	LC
20	*Dacrydium balansae* Brongn. and Gris	*Metadacrydium balansae*	-	New Caledonia	LC
21	*Dacrydium beccarii* Parl.	*Nageia beccarii*	-	Indonesia (Maluku, Papua, Sulawesi, Sumatera), Malaysia (Peninsular Malaysia, Sarawak), Papua New Guinea (Bismarck Archipelago), Philippines, Solomon Islands	LC
22	*Dacrydium comosum* Corner	*Corneria comosa*	-	Malaysia (Genting Highlands, Gunung Hulu Kali, Negeri Pahang)	EN
23	*Dacrydium cornwallianum* de Laub.	*Corneria cornwalliana*, *Dacrydium nidulum* var. *araucarioides*	-	Indonesia (Papua), Papua New Guinea	LC
24	*Dacrydium cupressinum* Sol. ex G.Forst.	*Dacrydium cupressiforme*, *Thalamia cupressina*	Rimu, Red pine	New Zealand	LC
25	*Dacrydium elatum* (Roxb.) Wall. ex Hook.	*Corneria elata*, *Corneria pierrei*, *Dacrydium beccarii* var. *subelatum*, *Dacrydium junghuhnii*, *Dacrydium pierrei*, *Juniperus elata*, *Juniperus elatus*, *Juniperus philippsiana*	Sempilor	Cambodia, Indonesia (Sumatera), Lao People’s Democratic Republic, Malaysia (Peninsular Malaysia, Sabah, Sarawak), Philippines, Thailand, Viet Nam	LC
26	*Dacrydium ericoides* de Laub.	*Corneria ericoides*	Sempilor, Bintulu	Malaysia (Borneo)	NA (IUCN) VU
27	*Dacrydium gibbsiae* Stapf	*Corneria gibbsiae*, *Dacrydium beccarii* var. *kinabaluense*	-	Malaysia (Mount Kinabalu in Sabah)	LC
28	*Dacrydium gracile* de Laub.	*Corneria gracilis*	-	Malaysia (Sabah, Sarawak)	NT
29	*Dacrydium guillauminii* J.Buchholz	*Gaussenia guillauminii*	-	New Caledonia (river Madeleine (Riviére des Lacs) and along the riverbanks of Lac en Huit)	CR
30	*Dacrydium leptophyllum* (Wasscher) de Laub.	*Bracteocarpus leptophyllus*, *Corneria lepto. phylla*, *Dacrycarpus leptophyllus*, *Dacrydium leptophyllum*, *Podocarpus leptophylla*, *Podocarpus leptophyllus*	-	Indonesia (Mt. Goliath, Papua)	VU
31	*Dacrydium lycopodioides* Brongn. and Gris	*Gaussenia lycopodioides*	Mou	New Caledonia (southern massif, from Mont Nekandi to Mont Dzumac and Mont Mou)	NT
32	*Dacrydium magnum* de Laub.	*Corneria magna*, *Dacrydium beccarii* var. *rudens*	-	Indonesia (Maluku), Papua New Guinea (Tagula Island, Normanby Island)	NT
33	*Dacrydium medium* de Laub.	*Corneria media*	Sangu, Gajo	Indonesia (Sumatera), Malaysia (Peninsular Malaysia)	VU
34	*Dacrydium nausoriense* de Laub.	*Corneria nausoriensis*	-	Fiji (Nausori Highlands, Sarava)	EN
35	*Dacrydium nidulum* de Laub.	*Corneria nidula*, *Dacrydium nidulum* var. *vitiensis*	-	Fiji, Indonesia (Lesser Sunda Is., Maluku, Papua, Sulawesi), Papua New Guinea	LC
36	*Dacrydium novoguineense* Gibbs	*Corneria novoguineensis*	-	Indonesia (Papua, Sulawesi), Papua New Guinea	LC
37	*Dacrydium pectinatum* de Laub.	*Corneria pectinata*, *Dacrydium pectinatum* var. *robustum*	-	Brunei Darussalam, China (Hainan), Indonesia (Kalimantan, Sumatera), Malaysia (Sabah, Sarawak), Philippines	EN
38	*Dacrydium spathoides* de Laub.	*Corneria spathoides*	-	Papua New Guinea (Irian Jaya), Indonesia	NT
39	*Dacrydium* × *suprinii* Nimsch	-	-	New Caledonia	NA
40	*Dacrydium xanthandrum* Pilg.	*Corneria xanthandra*	Kerapui	Indonesia (Kalimantan, Maluku, Papua, Sulawesi, Sumatera), Malaysia (Sabah, Sarawak), Papua New Guinea (Bismarck Archipelago, North Solomons), Philippines	LC
41	*Falcatifolium angustum* de Laub.	-	-	Malaysia (Sarawak)	EN
42	*Falcatifolium falciforme* (Parl.) de Laub.	*Podocarpus falciformis*, *Nageia falciformis*, *Falcatifolium usan-apuensis*, *Falcatifolium falciforme var. usan-apuensis*, *Falcatifolium falciforme var. kinabaluensis*, *Falcatifolium falciforme var. kinabaluensis*	-	Brunei Darussalam; Indonesia (Kalimantan); Malaysia (Peninsular Malaysia, Sabah, Sarawak)	NT
43	*Falcatifolium gruezoi* de Laub.	-	-	Indonesia (Maluku, Sulawesi), Philippines	NT
44	*Falcatifolium papuanum* de Laub.	*Dacrydium papuanum*	-	Papua New Guinea (Morobe)	LC
45	*Falcatifolium sleumeri* de Laub. and Silba	-	-	Indonesia (Papua)	NT
46	*Falcatifolium taxoides* (Brongn. and Gris) de Laub.	*Dacrydium taxoides*, *Nageia taxoides*, *Pinus falciformis*, *Podocarpus taxodioides*, *Podocarpus taxodioides* var. *gracilis*	-	New Caledonia	LC
47	*Halocarpus bidwillii* (Hook. f. ex Kirk) C.J.Quinn	*Dacrydium bidwillii*, *Dacrydium bidwillii* var. *erectum*, *Dacrydium bidwillii* var. *relinatum*	-	New Zealand (North Island, South Island, Stewart Island)	LC
48	*Halocarpus biformis* (Hook.) C.J.Quinn	*Dacrydium biforme*, *Podocarpus biformis*	Yellow pine, Pink pine, Bog Pine, Mountain Pine, Tarwood	New Zealand (North Island, South Island and Stewart Island)	LC
49	*Halocarpus kirkii* (F.Muell. ex Parl.) C.J.Quinn	*Dacrydium kirkii*	Monoao	New Zealand (Hokianga, Manukau Harbor and Coromandel Peninsula)	NT
50	*Lagarostrobos franklinii* (Hook.f.) Quinn	*Dacrydium franklinii*, *Lepidothamnus franklinii*	Huon Pine	Australia (Tasmania,)	LC
51	*Lepidothamnus fonkii* Phil.	*Dacrydium fonckii*, *Dacrydium fonckii*	Chilean Rimu	Argentina (Chubut, Santa Cruz); Chile (Aisén, Los Lagos, Magellanes)	LC
52	*Lepidothamnus intermedius* (Kirk) Quinn	*Dacrydium intermedium*	Yellow silver pine, Mountain pine	New Zealand (South Island, Stewart Island and North Island)	LC
53	*Lepidothamnus laxifolius* (Hook.f.) Quinn	*Dacrydium laxifolium*, *Dacrydium laxifolium* var. *compactum*, *Dacrydium laxifolium* var. *debile*	Mountain rimu, Pigmy pine, Pygmy pine	New Zealand (Tongariro National Park in the North Island southwards to Stewart Island)	LC
54	*Manoao colensoi* (Hook.) Molloy	*Dacrydium colensoi*, *Dacrydium westlandicum*, *Lagarostrobos colensoi*	Manoao, Silver pine, Westland pine, White silver pine	New Zealand (Lake Te Anau, Central Volcanic Plateau and Auckland)	LC
55	*Microcachrys tetragona* (Hook.) Hook.f.	*Dacrydium tetragonu*, *Athrotaxis tetragona*	Strawberry pine, Creeping Pine,	Australia (Tasmania)	LC
56	*Nageia fleuryi* (Hickel) de Laub.	*Decussocarpus fleuryi*, *Podocarpus fleuryi*	Kim giao	China (Guangdong, Guangxi, Yunnan), Laos, Vietnam	NT
57	*Nageia formosensis* (Dummer) C.N.Page	*Decussocarpus nagi* var. *formosensis*, *Nageia nagi* var. *formosensis*, *Nageia nagi* var. *koshuensis*, *Nageia nankoensis*, *Podocarpus formosensis*, *Podocarpus formosensis var. koshuensis*, *Podocarpus koshunensis*, *Podocarpus nagi var. koshuensis*	-	Taiwan	NA
58	*Nageia maxima* (de Laub.) de Laub.	*Decussocarpus maximus*, *Podocarpus maxima*, *Podocarpus maximus*	Landin paya	Malaysia (Sarawak)	EN
59	*Nageia motleyi* (Parl.) de Laub.	*Agathis motleyi*, *Dammara motleyi*, *Decussocarpus motleyi*, *Nageia baccarii*, *Podocarpus beccarii*, *Podocarpus motleyi*	Podo kebal musang, Kayu bawa, Setebal, Medang buloh	Brunei Darussalam, Indonesia (Kalimantan, Sumatera), Malaysia (Peninsular Malaysia, Sabah, Sarawak), Thailand	VU
60	*Nageia nagi* (Thunb.) Kuntze	*Myrica nagi*, *Agathis veitchii*, *Dammara veitchii*, *Decussocarpus nagi*, *Nageia caesia*, *Nageia cuspidata*, *Nageia grandifolia*, *Nageia ovata*, *Podocarpus caesius*, *Podocarpus cuspidatus*, *Podocarpus grandifolius*, *Podocarpus nageia*, *Podocarpus nageia* var. *angustifolius*, *Podocarpus nageia* var. *rotundifolius*, *Podocarpus nagi*, *Podocarpus nagi* var. *angustifolius*, *Podocarpus nagi* var. *caesius*, *Podocarpus nagi* var. *ovatus*, *Podocarpus nagi* var. *rotundifolius*, *Podocarpus ovata*, *Podocarpus ovatus*	Broad-leaved podocarp	Introduced China (Fujian, Guangdong, Guangxi, Hainan, Hunan, Jiangxi, Sichuan, Zhejiang), Japan (Honshu, Kyushu, Nansei-shoto, Shikoku), Taiwan (introduced), Vietnam (Lang Son)	NT
61	*Nageia wallichiana* (C.Presl) Kuntze	*Decussocarpus wallichianus*, *Nageia blumei*, *Podocarpus blumei*, *Podocarpus wallichianus*	Mala almaciga	Brunei Darussalam, Cambodia, China (Yunnan), India (Assam, Kerala, Nicobar Island), Indonesia (Jawa, Kalimantan, Maluku, Papua, Sulawesi, Sumatera), Laos, Malaysia (Peninsular Malaysia, Sabah, Sarawak), Myanmar, Papua New Guinea, Philippines, Thailand, Vietnam	LC
62	*Parasitaxus usta* (Vieill.) de Laub.	*Dacrydium ustum*, *Nageia usta*, *Parasitaxus usta*, *Podocarpus ustus*	Corail, Cèdre rabougri	New Caledonia [Grand Terre in the mountains of the far south (Dzumac, Montagne des Sources), central west (Paéoua and Tchingou) and far northeast (Colnett/Panie)]	VU
63	*Pherosphaera fitzgeraldii* (F.Muell.) Hook.f.	*Dacrydium fitzgeraldii*, *Microstrobos fitzgeraldii*	Dwarf mountain pine, Blue Mountain dwarf pine	Australia (New South Wales)	CR
64	*Pherosphaera hookeriana* W.Archer bis	*Dacrydium hookerianum* (W.Archer bis) Eichler, *Microstrobos niphophilus* J.Garden and L.A.S.Johnson, *Pherosphaera niphophila* (J.Garden and L.A.S.Johnson) Florin	Drooping pine, Mount Mawson Pine	Australia (Tasmania)	NT
65	*Phyllocladus aspleniifolius* (Labill.) Hook.f.	*Brownetera aspleniifolia* (Labill.) Tratt., *Phyllocladus glaucus* Carrière, *Phyllocladus rhomboidalis* Rich., *Phyllocladus serratifolius* Nois. ex Henkel and Hochst., *Phyllocladus trichomanoides* var. *glaucus* (Carrière) Parl., *Podocarpus aspleniifolius* Labill., *Thalamia asplenifolia* (Labill.) Spreng.	Celery top pine	Australia (Tasmania)	LC
66	*Phyllocladus hypophyllus* Hook.f.	*Phyllocladus hypophyllus* var. *protractus* Warb., *Phyllocladus major* Pilg., *Phyllocladus protractus* (Warb.) Pilg., *Podocarpus hypophyllus* (Hook.f.) Kuntze	Celery top pine	Philippines, Brunei, Malaysia (Celebes, Moluccas, Sulawesi), Papua New Guinea, Indonesia (Maluku)	LC
67	*Phyllocladus toatoa* Molloy	*Phyllocladus glaucus* Kirk	Toatoa (Maori), Blue celery pine	New Zealand (North Island)	LC
68	*Phyllocladus trichomanoides* D.Don	*Phyllocladus cunninghamii*, *Podocarpus trichomanoides*	Tanekaha (Maori), Celery pine	New Zealand (North Island and South Island)	LC
69	*Phyllocladus trichomanoides* var. *alpinus* (Hook.f.) Parl.	*Phyllocladus alpinus*, *Phyllocladus aspleniifolius* var. *alpinus*	Mountain toatoa	New Zealand	LC
70	*Podocarpus acuminatus* de Laub.	-	-	Brazil (Serra da Neblina in the state of Amazonas), Venezuela (Bolívar)	NT
71	*Podocarpus acutifolius* Kirk	*Nageia acutifolia*	Needle-leaved totara, Westland totara	New Zealand (South Island from Marlborough Sounds to S Westland)	LC
72	*Podocarpus affinis* Seem.	*Nageia affinis*	Kuasi	Fiji (Higher ridges on Viti Levu)	NT
73	*Podocarpus angustifolius* Griseb.	*Podocarpus victorinianus*, *Podocarpus leonii*, *Podocarpus aristulatus*	Sabina cimarrona	Cuba Cienfuegos (Sierra de Trinidad), Guantánamo (Sierra Maestra), Sancti Spíritus (Sierra de Sancti Spíritu), Holguín and Santiago de Cuba (Sierra de Nipe, Sierra del Cristal, and the Baracoa Ranges).	VU
74	*Podocarpus annamiensis* N.E.Gray	*-*	-	Myanmar, Vietnam, China	TH
75	*Podocarpus aracensis* de Laub. and Silba	-	-	Brazil [Amazonas (Serra Araca, Cerro Neblina] and Venezuela [Amazonas (Cerro Yaví)]	LC
76	*Podocarpus archboldii* N.E. Gray	*Margbensonia archboldi*, *Podocarpus crassigemma*	Baula, Jamekang, juba, Kaibigltuga, Morumba, Puling, Yamekange, Mu, Soa, Sarau	Indonesia (Papua), Papua New Guinea (Morobe District0	VU
77	*Podocarpus aristulatus* Parl.	*Nageia aristulata*, *Podocarpus angustifolius* var. *wrightii*		Cuba, Dominican Republic,	TH
78	*Podocarpus atjehensis* (Wasscher) de Laub.	*Podocarpus neriifolius* var. *atjehensis*, *Margbensonia atjehensis*, *Margbensonia atjehense*, *Podocarpus neriifolius* var. *membranaceaus*	Atjeh	Malesia [Sumatera (Aceh, Gajo Lands, Kemiri and Bandahara), New Guinea (Papua, Wissel Lakes).	NT
79	*Podocarpus barretoi* de Laub. and Silba	-	-	Brazil (Minas Gerais)	NT
80	*Podocarpus beecherae* de Laub.	-	-	New Caledonia	TH
81	*Podocarpus borneensis* de Laub.	*Podocarpus polystachyus* var. *rigidus*	Bisit, Bubung, Buloh	Indonesia (Kalimantan), Malaysia (Sabah, Sarawak)	LC
82	*Podocarpus bracteatus* Blume	*Nageia bracteata*, *Podocarpus bracteatus* var. *brevipes*, *Podocarpus neriifolius* var. *bracteatus*, *Podocarpus neriifolius* var. *brevipes*	Kayu unung unung, Toba Batak, Bima, Kimarak, Kipantjar, Ki putri	Indonesia (Jawa, Lesser Sunda Is., Sumatera)	LC
83	*Podocarpus brasiliensis* de Laub.	*Podocarpus barretoi*	-	Brazil (Bahia, Brasília Distrito Federal, Goiás, Mato Grosso, Minas Gerais, Roraima), Venezuela	LC
84	*Podocarpus brassii* Pilg.	-	Baugwa, Baula, Chuga, Kaibigltuga, Kaipil, Karbuku, Maja, Mbagua, Tsula	Indonesia (Papua), Papua New Guinea	LC
85	*Podocarpus brassii* var. *humilis* de Laub.	*Podocarpus brassii* subsp. *humilis*	-	Papua New Guinea	LC
86	*Podocarpus brevifolius* (Stapf) Foxw.	*Podocarpus neriifolius*	-	Malaysia (Sabah)	NT
87	*Podocarpus buchholzii* de Laub.	*Podocarpus buchholzii* var. *neblinensis* Silba, *Podocarpus buchholzii* subsp. *neblinensis* (Silba) Silba	-	Venezuela (Guyana Highlands)	LC
88	*Podocarpus buchii* Urb.	*Podocarpus aristulatus* var. *buchii*, *Podocarpus angustifolius* Griseb. ssp. *buchii*	Tachuela, Chicharrón, Palo de Cruz	Dominican Republic (Southeast Haiti)	EN
89	*Podocarpus capuronii* de Laub.	*Podocarpus capuronii* var. *capuronii*, *Podocarpus woltzii*	-	Madagascar (Itremo Massif, Manandona)	EN
90	*Podocarpus celatus* de Laub.	-	Cinqui-mase	Bolivia (Potosi), Brazil (Goiás, Mato Grosso), Colombia, Ecuador, Peru (Junin, Loreto, Montana, Puno), Venezuela (Amazonas, Bolivar, Tachira)	LC
91	*Podocarpus chinensis* Wall. ex J.Forbes	*Podocarpus macrophyllus* var. *maki*, *Podocarpus japonicus*, *Podocarpus makoyi*, *Podocarpus appressus*, *Podocarpus macrophyllus* subsp. *maki*, *Podocarpus maki*, *Nageia appressa*, *Nageia japonica*, *Nageia chinensis*, *Nageia macrophylla* var. *maki*, *Myrica esquirolii*	-	China (Anhui, Fujian, Guangdong, Guangxi, Guizhou, Hubei, Hunan, Jiangsu, Jiangxi, Shaanxi, Sichuan, Yunnan, and Zhejiang), Myanmar, Japan	LC
92	*Podocarpus chinensis* var. *wardii* de Laub. and Silba	*Podocarpus chinensis* subsp. *wardii*	-	Myanmar (N’mai Hka Valley)	LC
93	*Podocarpus chingianus* S.Y.Hu	*Podocarpus macrophyllus* var. *chingiiI*, *Margbensonia chingiana*	Zhu guan luo han song	China (Jiangsu, Zhejiang, Sichuan)	DD
94	*Podocarpus confertus* de Laub.	*Podocarpus neriifolius* var. *penibukanensis*	-	Malaysia (Sabah, Sarawak)	EN
95	*Podocarpus coriaceus* Rich. and A.Rich.	*Nageia coriacea*, *Taxus lancifolia*, *Podocarpus coriaceus* var. *sulcatus*	Yucca plum pine, Resinier moutaigue, Caoba del país	Dominican Republic, Guadeloupe, Martinique, Montserrat, Puerto Rico, Saint Kitts and Nevis, Saint Lucia, Trinidad, and Tobago	LC
96	*Podocarpus costalis* C.Presl	*Nageia costalis*, *Podocarpus costalis* var. *taiwanensis*	Lan yu luo han song, Arius	Philippines, Taiwan, China	EN
97	*Podocarpus costaricensis* de Laub.	-	Cipresillo	Costa Rica (San José)	CR (VU)
98	*Podocarpus crassigemma* de Laub.	*Podocarpus crassigemmis*	A-pul, Kaboga, Morumba, Baula, Iamuka, Jamekang, Juba, Kamga, Puling, Kabor, Kabiltugl, Kaibelparu, Kkaibig, Kaip, Nonofan, Ronohanini, Sula	Indonesia (Papua), Papua New Guinea (Bismarck Archipelago, Central highlands)	LC
99	*Podocarpus decipiens* N.E.Gray	*-*	-	Fiji (Viti Levu)	NA
100	*Podocarpus decumbens* N.E.Gray	-	-	New Caledonia (Grande Terre, Southern mountains)	CR
101	*Podocarpus deflexus* Ridl.	-	-	Indonesia (Sumatera), Malaysia (Peninsular Malaysia)	EN
102	*Podocarpus degeneri* (N.E.Gray) de Laub.	*Margbensonia degeneri*, *Podocarpus neriifolius* var. *degeneri*	-	Fiji	LC
103	*Podocarpus dispermus* C.T.White	*Margbensonia disperma*	Broad-leaved brown pine	Australia (Queensland)	LC
104	*Podocarpus drouynianus* F.Muell.	*Nageia drouyniana*, *Margbensonia drouyniana*, *Podocarpus brownii*	Emu Berry	Australia (Western Australia)	LC
105	*Podocarpus ekmanii* Urb.	-	Sabina Cimarrona	Cuba (Sierra del Cristal, Sierra de Moa and Sierra de Nipe)	LC
106	*Podocarpus elatus* R.Br. ex Endl	*Margbensonia elata*, *Nageia elata*	Illawarra plum, Brown pine, Plum pine, Turpentine pine, Yellow pine, Australian plum, White plum, Goongum, Native deal, Pencil cedar	Australia (New South Wales, Queensland)	LC
107	*Podocarpus elongatus* (Aiton) L’Hér. ex Pers.	*Taxus elongatus*, *Taxus elongata*, *Taxus capensis*, *Nageia elongata*, *Podocarpus thunbergii* var. *angustifolia*	Breede river yellowwood	Malawi, South Africa (Northern Cape Province, Western Cape), Zambia, Zimbabwe	LC
108	*Podocarpus fasciculus* de Laub.	*Podocarpus macrophyllus* var. *liukiuensis*, *Podocarpus macrophyllus* f. *grandifolia*	-	Japan (Nansei-shoto), Taiwan	VU
109	*Podocarpus forrestii* Craib and W.W.Sm.	*Margbensonia forrestii*, *Podocarpus macrophyllus* subsp. *forrestii*	-	China	VU
110	*Podocarpus gibbsiae* N.E.Gray	-	-	Malaysia (Endemic to Mt. Kinabalu in Sabah)	VU
111	*Podocarpus glaucus* Foxw.	-	Nipa	Indonesia (Maluku, Papua, Sulawesi), Papua New Guinea (Bismarck Archipelago), Philippines, Solomon Islands	LC
112	*Podocarpus globulus* de Laub.	-	Sapiro	Malaysia (Sabah, Sarawak)	EN
113	*Podocarpus glomeratus* D.Don	*Nageia glomerata*, *Podocarpus rigidus*, *Podocarpus cardenasii*	Pino de Monte, Intimpa, Huampo	Bolivia, Ecuador, Peru	NT
114	*Podocarpus gnidioides* Carrière	*Nageia gnidioides*, *Podocarpus alpinus* var. *arborescens*, *Podocarpus alpinus* var. *caespitosus*, *Podocarpus caespitosus*, *Podocarpus gnidioides* subsp. *caespitosus*	-	New Caledonia	NT
115	*Podocarpus grayae* de Laub.	-	Brown pine, Northern brown pine; Brown pine; Weeping brown pine	Australia (Northern Territory, Queensland)	LC
116	*Podocarpus guatemalensis* Standl.	*Podocarpus allenii*, *Podocarpus guatemalensis* var. *allenii*, *Podocarpus guatemalensis* subsp. *allenii*, *Podocarpus guatemalensis* subsp. *pinetorum*, *Podocarpus guatemalensis* var. *pinetorum*, *Podocarpus pinetorum*	Ocotillo de Llano, Alfajillo, Ciprecillo Amarillo, Ciprecillo Blanco, Cipresillo, Cypress de Montaña, Palo de Oro, Pinillo, Piño de Montaña	Belize, Colombia, Costa Rica, Ecuador, El Salvador, Guatemala, Honduras, Mexico (Oaxaca, Veracruz), Nicaragua, Panama	LC
117	*Podocarpus henkelii* Stapf ex Dallim. and B.D.Jacks.	*Podocarpus ensiculus*, *Podocarpus henkelii* subsp. *Ensiculus*, *Podocarpus thunbergii* var. *falcata*	Henkel’s yellowwood, Falcate yellowwood, East griqualand yellowwood, Natal yellowwood, bastergeelhout, Nanjula	Malawi, South Africa (Eastern Cape Province, KwaZulu-Natal), Tanzania, Zimbabwe	CR
118	*Podocarpus hispaniolensis* de Laub.	-	-	Dominican Republic (Cordillera Central, San José de Ocoa, Cordillera Septentriona, Province Puerto Plata)	EN
119	*Podocarpus hookeri* de Laub.	*Podocarpus neriifolius* var. *linearis*, *Podocarpus neriifolius* var. *staintonii*	-	India (Sikkim), Indonesia (Sumatra, Java, Borneo), Philippines	LC
120	*Podocarpus humbertii* de Laub.	-	-	Madagascar (Mont Anjanaharibe, Mont Tsaratanana and Mont Marojezy)	EN
121	*Podocarpus insularis* de Laub.	-	Dala, tunum, ida-ayebo	Papua New Guinea (Bismarck Archipelago), Solomon Islands, Vanuatu	LC
122	*Podocarpus idenburgensis* N.E.Gray	-	-	Papua New Guinea, Fiji	NE
123	*Podocarpus laetus* Hooibr. ex Endl.	*Podocarpus cunninghamii*, *Podocarpus hallii*, *Nageia hallii*, *Podocarpus totara* var. *hallii*	Montane totara, Thin-bark totara, Hall’s totara	New Zealand (North Island, Tongariro National Park, South Island and Stewart Island)	LC
124	*Podocarpus lambertii* Klotzsch ex Endl.	*Nageia lambertii*, *Podocarpus lambertii* subsp. *horsmanii*, *Podocarpus lambertii* var. *horsmanii*, *Podocarpus lambertii* subsp. *tigreensis*, *Podocarpus lambertii* var. *tigreensis*	Pinheiro bravo	Argentina (Misiones), Brazil (Minas Gerais, Paraná, Rio de Janeiro, Rio Grande do Sul, Santa Catarina, São Paulo)	NT
125	*Podocarpus laminaris* de Laub.	*Podocarpus rubens* subsp. *pabinamaensis*, *Podocarpus rubens* var. *pabinamaensis*	-	Papua New Guinea	NA
126	*Podocarpus latifolius* (Thunb.) R.Br. ex Mirb.	*Nageia latifolia*, *Nageia thunbergii*, *Podocarpus latifolius var. latior*, *Podocarpus latifolius subsp. latior*, *Podocarpus latior*, *Podocarpus nobilis*, *Podocarpus pinnata*, *Podocarpus thunbergii*, *Taxus latifolia*	Broad-leaved yellowwood, Real yellowwood, True yellowwood, Upright yellowwood	Eswatini, South Africa (Eastern Cape Province, Free State, KwaZulu-Natal, Limpopo Province, Mpumalanga, Northern Cape Province, Western Cape)	LC
127	*Podocarpus laubenfelsii* Tiong	-	-	Indonesia (Kalimantan), Malaysia (Sabah, Sarawak)	EN
128	*Podocarpus lawrencei* Hook.f.	*Podocarpus alpinus* var. *lawrencei*, *Podocarpus lawrencei* subsp. *errinundraensis*	Mountain plum pine, Plum pine	Australia (Australian Capital Territory, New South Wales, Tasmania, Victoria)	LC
129	*Podocarpus ledermannii* Pilg.	*Podocarpus idenburgensis*	-	Indonesia (Papua), Papua New Guinea (Bismarck Archipelago)	LC
130	*Podocarpus ledermannii* Pilg. var. *expansus* de Laub.	-	-	Indonesia	LC
131	*Podocarpus lenticularis* de Laub.	-	-	Assam (India), Laos	NA
132	*Podocarpus linearis* de Laub.	-	-	Papua New Guinea	DD
133	*Podocarpus levis* de Laub.	-	Marisa, Sanru, Kayu tjina, Wasiwarare	Indonesia (Kalimantan, Maluku, Papua, Sulawesi)	LC
134	*Podocarpus × loderi* Cockayne	-	-	New Zealand	NA
135	*Podocarpus longifoliolatus* Pilg.	*Podocarpus longifoliolatus*	-	New Caledonia (Grande Terre)	EN
136	*Podocarpus lophatus* de Laub.	-	-	Philippines (Mt. Tapulao in Luzon and Mt. Halcon in Mindoro)	VU
137	*Podocarpus lucienii* de Laub.	-	-	New Caledonia (Massif du Colnett and the Massif du Tchingou)	LC
138	*Podocarpus macrocarpus* de Laub.	-	Malakawayan	Philippines (Luzon)	EN
139	*Podocarpus macrophyllus* (Thunb.) Sweet	*Margbensonia forrestii*, *Margbensonia macrophylla*, *Nageia macrophylla*, *Nageia macrophylla*, *Podocarpus forrestii*, *Podocarpus macrophyllus* var. *angustifolius*, *Podocarpus macrophyllus* subsp. *angustifolius*, *Podocarpus macrophyllus* f. *angustifolius*, *Podocarpus macrophyllus* subsp. *forrestii*, *Podocarpus macrophyllus* var. *macrophyllus*, *Podocarpus macrophyllus* var. *rubra*, *Podocarpus verticillatus*, *Taxus macrophylla*, *Taxus makoya*	Southern yew, Yew podocarp, Long-leaved podocarp, Buddhist pine, Kusamaki, Inumaki, luo han song	China (Anhui, Chongqing, Fujian, Guangxi, Guizhou, Hubei, Hunan, Jiangsu, Jiangxi, Sichuan, Yunnan, Zhejiang), Hong Kong, Japan (Honshu, Kyushu, Shikoku), Taiwan, Malaysia, Singapore	LC
140	*Podocarpus macrophyllus* var. *piliramulus* Zhi X. Chen and Zhen Q. Li	-	-	China (Anhui, Chongqing, Fujian, Guangdong, Guangxi, Guizhou, Hunan, Jiangxi, Yunnan, Zhejiang); Hong Kong; Japan (Honshu, Kyushu, Shikoku); Myanmar; Taiwan	NT
141	*Podocarpus madagascariensis* Baker	Nageia madagascariensis, Podocarpus madagascariensis var. madagascariensis	-	Madagascar	NT
142	*Podocarpus madagascariensis* var. *procerus* de Laub.	*Podocarpus madagascariensis* subsp. *procerus*	-	Madagascar (Tolanaro [Fort Dauphin] and Massif de Bekolosy).	EN
143	*Podocarpus madagascariensis* var. *rotundus* L.Laurent	*Podocarpus madagascariensis* subsp. *rotundus* (L. Laurent) Silba	-	Madagascar (Massif de Bekolosy and the Massif du Manongarivo)	DD
144	*Podocarpus magnifolius* J.Buchholz and N.E. Gray	-	Cinqui-masé	Bolivia (La Paz), Colombia, Panama, Peru (Pasco, Oxapampa), Venezuela (States of Bolívar, Amazonas, Aragua, Yaracuy)	LC
145	*Podocarpus marginalis* de Laub.	-	-	Papua New Guinea	DD
146	*Podocarpus matudae* Lundell	*Podocarpus reichei*, *Podocarpus matudae* var. *reichei*, *Podocarpus matudae* var. *macrocarpus*, *Podocarpus matudae* var. *jaliscanus*, *Podocarpus matudae* subsp. *jaliscanus*, *Podocarpus matudae* subsp. *macrocarpus*, *Podocarpus matudae* subsp. *reichei*	Sabino	Mexico (Chiapas, Jalisco, Michoacán, Nayarit, Oaxaca, Puebla, Querétaro, San Luis Potosí, Tamaulipas, Veracruz), El Salvador, Guatemala (Huehuetenango), Honduras	CR
147	*Podocarpus matudae* subsp. *jaliscanus* (de Laub. and Silba) Silba	*Podocarpus matudae* Lundell var. *jaliscanus*	-	Mexico (Jalisco)	VU
148	*Podocarpus micropedunculatus* de Laub.	-	Kayu china, kayu tjina	Brunei Darussalam, Indonesia, Malaysia (Sabah, Sarawak)	NT
149	*Podocarpus milanjianus* Rendle	*Podocarpus ulugurensis*	Lusamina	Angola, Burundi, Cameroon, Congo, Congo, Kenya, Malawi, Mozambique, Nigeria, Rwanda, Sudan, Tanzania, Uganda, Zambia, Zaire, Zimbabwe	LC
150	*Podocarpus nakaii* Hayata	*Podocarpus macrophyllus* var. *nakaii*	Nakai podocarp, Nakai yellowwood	Taiwan (Chianghua Co., Nantou Co., Taichung Co.)	EN
151	*Podocarpus neriifolius* D.Don	*Nageia discolor*, *Nageia leptostachya*, *Nageia neriifolia*, *Nageia neglecta*, *Nageia decipiens*, *Nageia polyantha*, *Nageia annamiensis*, *Podocarpus discolor*, *Podocarpus leptostachya*, *Podocarpus annamiensis*, *Podocarpus epiphyticus*, *Podocarpus neglecta*, *Podocarpus junghuhniana*, *Podocarpus thailandensis*, *Podocarpus neriifolius* var. *polyanthus*	Brown pine, Podo bukit, Ambai Ayam, Hatang, Hai nan luo han song, Thông tre, Thông lông gà	Brunei Darussalam, Cambodia, China (Guangxi, Yunnan), Fiji, India (Assam, West Bengal), Bangladesh, Indonesia (Bali, Jawa, Kalimantan, Lesser Sunda Is., Maluku, Papua, Sulawesi, Sumatera), Lao People’s Democratic Republic, Malaysia (Peninsular Malaysia, Sabah, Sarawak); Myanmar, Nepal, Papua New Guinea (Bismarck Archipelago), Philippines, Solomon Islands, Thailand, Vietnam	LC
152	*Podocarpus neriifolius* D.Don var. *degeneri* N.E.Gray	-	-	Fiji (Vanua Leva, Viti Levu)	LC
153	*Podocarpus nivalis* Hook.	*Nageia nivalis*, *Podocarpus nivalis* var. *erectus*, *Podocarpus montanus*	Alpine totara, Snow totara	New Zealand (North Island, South Island)	LC
154	*Podocarpus novae-caledoniae* Vieill. ex Brongn. and Gris	*Nageia novae-caledoniae*, *Podocarpus beecherae*, *Podocarpus rivularis*	-	New Caledonia (Grande Terre, Iledes Pins)	LC
155	*Podocarpus neglectus* Blume	*Podocarpus discolor*, *Podocarpus leptostachyus*, *Podocarpus thailandensis*, *Nageia neglecta*, *Podocarpus discolor*, *Podocarpus junghuhnianus*	-	Thailand, Indonesia, China (Hainan), Indonesia (Borneo, Java, Sumatra), Malaysia	NA
156	*Podocarpus novoguineensis* de Laub.	-	-	Papua New Guinea	NA
157	*Podocarpus nubigenus* Lindl.	*Nageia nubigena*, *Saxegothaea gracilis*	Huililahuani, Mañio, Mañío de Hojas Punzantes, Mañio Hembra, Mañio Macho, Mañiu de la Costa, Pino Amarillo	Argentina (Neuquén, Santa Cruz), Chile (Aisén, La Araucania, Los Lagos, Magellanes)	NT
158	*Podocarpus oblongus* de Laub.	*Podocarpus rumphii* subsp. *Aruensis*, *Podocarpus rumphii* var. *aruensis*	-	Papua New Guinea (Vogelkop)	DD
159	*Podocarpus oleifolius* D.Don	*Nageia macrostachya*, *Nageia oleifolia*, *Podocarpus ballivianensis*, *Podocarpus ingensis*, *Podocarpus macrostachys*, *Podocarpus oleifolius* subsp. *equadorensis*, *Podocarpus oleifolius* var. *equadorensis*, *Podocarpus oleifolius* var. *macrostachys*, *Podocarpus oleifolius* var. *trujillensis*, *Podocarpus oleifolius* subsp. *trujillensis*	Pino de pasto, Pinete	Bolivia, Colombia, Costa Rica, Ecuador, El Salvador, Guatemala, Honduras, Mexico (Chiapas, Guerrero, Michoacán, Oaxaca, Puebla, Veracruz), Panama, Peru, Venezuela	LC
160	*Podocarpus oleifolius* subsp. *costaricensis* (J.Buchholz and N.E.Gray) Silba	-	-	Costa Rica, El Salvador, Guatemala, Honduras, Mexico Gulf, Mexico Southeast, Mexico Southwest, Nicaragua, Panamá	LC
161	*Podocarpus orarius* R.R.Mill and M.Whiting	*Podocarpus spathoides* subsp. *solomonensis*, *Podocarpus spathoides* var. *solomonensis*	-	Papua New Guinea (Solomon Island), Vanuatu	NT
162	*Podocarpus palawanensis* de Laub. and Silba	-	-	Philippines (Palawan)	CR
163	*Podocarpus pallidus* N.E.Gray	-	Uhiuhi	Tonga (island of Eua and islands of Vava’u)	VU
164	*Podocarpus parlatorei* Pilg.	*Podocarpus angustifolius*, *Nageia angustifolia*	Pino Blanco, Pino del Cerro	Argentina (Catamarca, Corrientes, Jujuy, Salta, Tucumán), Bolivia (Chuquisaca, Cochabamba, Potosí, Santa Cruz, Tarija, La Paz), Peru (Sierra de Chaglla)	NT
165	*Podocarpus pendulifolius* J.Buchholz and N.E.Gray	-	Pino Carbón, Pino Hayuco	Venezuela (Andes, Cordillera do Merida, Edo Lara, Merida, Tachira and Trujillo)	EN
166	*Podocarpus perrieri* Gaussen and Woltz	*Podocarpus rostratus* var. *perrieri*, *Podocarpus rostratus* subsp. *perrieri*	-	Madagascar (Andringitra Massif, Toamasina, Forêt d’Andasibé)	CR
167	*Podocarpus pilgeri* Foxw.	*Podocarpus celebicus*, *Podocarpus pilgeri* var. *thailandensis*, *Podocarpus pilgeri* subsp. *wangii*, *Podocarpus schlechteri*, *Podocarpus wangii*, *Podocarpus tixieri*	-	Cambodia (Kampuchea), China (Guangdong, Guangxi, Hainan, Yunnan), Indonesia (Maluku, Papua, Sulawesi), Laos, Malaysia (Sarawak), Papua New Guinea (Bismarck Archipelago); Philippines, Thailand, Vietnam	LC
168	*Podocarpus polyspermus* de Laub.	-	Mé Maoya podocarp	New Caledonia (Grande Terre)	EN
169	*Podocarpus polystachyus* R.Br. ex Endl.	*Margbensonia polystachya*, *Nageia polystachya*	Jati bukit, Kayu karamat, Podo laut, Mayu serai, Landin, Kandabang, kayu china, Saumah	Brunei Darussalam, Indonesia (Kalimantan, Maluku, Papua, Sumatera), Malaysia (Peninsular Malaysia, Sabah, Sarawak), Papua New Guinea, Philippines, Singapore, Thailand	VU
170	*Podocarpus pseudobracteatus* de Laub.	*Podocarpus archboldii* var. *crassiramosus*	Kaip, Kebu, Puling	Indonesia (Papua), Papua New Guinea	LC
171	*Podocarpus pseudobracteatus* de Laub. var. *sicaris* de Laub.	-	-	Papua New Guinea	LC
172	*Podocarpus purdieanus* Hook.	*Nageia purdieana*	Yacca, St. Ann Yacca	Jamaica (Claredon, St. Catherine, St. Ann, Trelawny, Sanders Hill, Mt. Diablo)	EN
173	*Podocarpus ramosii* R.R.Mill	*Podocarpus rotundus*	-	Indonesia (Kalimantan Timur), Philippines (Mt. Banahao in Luzon)	DD
174	*Podocarpus ridleyi* (Wasscher) N.E. Gray	*Margbensonia ridleyi*, *Podocarpus neriifolius* var. *ridleyi*	-	Malaysia (Peninsular Malaysia)	VU
175	*Podocarpus roraimae* Pilg.	*Podocarpus buchholzii*, *Podocarpus buchholzii* var. *neblinensis*, *Podocarpus buchholzii* subsp. *neblinensis*	Ai-yek	Guyana (Region of Cuyuni-Mazaruni on Mt. Roraima), Venezuela (Amazonas, Bolívar)	LC
176	*Podocarpus rostratus* J.Laurent	-	-	Madagascar (Antsiranana, Fianarantsoa, Mahajanga and Toamasina Provinces)	EN
177	*Podocarpus rubens* de Laub.	*Podocarpus indonesiensis*, *Podocarpus rubens* subsp. *sumatranus*, *Podocarpus rubens* var. *sumatranu*, *Podocarpus rubens* var. *pabinamaensis*, *Podocarpus neriifolius* var. *timorensis*	Bebi-è, Ungpop, Bin, Kaip, Nelil, Sukou	Indonesia (Maluku, Flores, Borneo, Sulawesi, Sumatera), Malaysia (Sabah), Papua New Guinea (Bismarck Archipelago, Papuasia, New Britain), Timor-Leste	LC
178	*Podocarpus rumphii* Blume	*Cerbera nereifolia*, *Margbensonia koordersii*, *Margbensonia philippinensis*, *Margbensonia rumphii*, *Nageia rumphii*, *Podocarpus koordersii*, *Podocarpus philippinensis*, *Podocarpus rumphii* subsp. *arbainii*, *Podocarpus rumphii* var. *arbainii*, *Podocarpus sprengelii*, *Podocarpus rumphii* var. *aruensis*	Nimsal	China (Hainan), Indonesia (Jawa, Lesser Sunda Island, Maluku, Papua, Sulawesi), Malaysia (Peninsular Malaysia, Sabah), Papua New Guinea (Bismarck Archipelago), Philippines (Luzon)	NT
179	*Podocarpus rusbyi* J.Buchholz and N.E.Gray	-	Pino Blanco, Pino del Monte	Bolivia (Cochabamba, La Paz, Santa Cruz), Peru (Cusco and near Machu Pichu)	VU
180	*Podocarpus salicifolius* Klotzsch and H.Karst. ex Endl.	*Nageia salicifolia*, *Podocarpus pittieri*	Pinabete	Venezuela, Brazil, Bolivia, Colombia, Peru	LC
181	*Podocarpus salignus* D. Don	*Nageia chilina*, *Podocarpus chilinus*, *Podocarpus chilinus* var. *glaucus*	Willow-leaf podocarp, Mañio, Mañío de Hojas Largas, Mañio de Hojas Punzantes, Mañio Hembra, Mañio Macho, Manique, Pino Amarillo	Chile (Biobío, La Araucania, Los Lagos, Maule)	VU
182	*Podocarpus salomoniensis* Wasscher	-	Dengali tolo	Solomon Islands (San Cristobal Island and San Jorge Island)	EN
183	*Podocarpus sellowii* Klotzsch ex Endl.	*Nageia sellowii*	-	Brazil (Paraná, Rio de Janeiro, Rio Grande do Sul, Santa Catarina, São Paulo)	EN
184	*Podocarpus sellowii* Klotzch ex Endl. var. *angustifolius*	-	-	Brazil (Rio de Janeiro)	CR
185	*Podocarpus smithii* de Laub.	-	Smith’s pine, Brown pine	Australia (Queensland)	LC
186	*Podocarpus spathoides* de Laub.	*Podocarpus spathoides* de Laub. var. *solomonensis*	-	Malaysia (Peninsular Malaysia, Sarawak, Maluku), Papua New Guinea (Solomon Islands)	DD
187	*Podocarpus spinulosus* (Sm.) R.Br. ex Mirb.	*Margbensonia spinulosa*, *Nageia ensifolia*, *Nageia laeta*, *Nageia spinulosa*, *Podocarpus bidwillii*, *Podocarpus ensifolius*, *Podocarpus laetus*, *Podocarpus pungens*, *Taxus spinulosa*	-	Australia (New South Wales, Queensland)	LC
188	*Podocarpus sprucei* Parl.	*Nageia sprucei*	Guabisay, Romerillo	Ecuador, Peru (Piura)	EN
189	*Podocarpus steyermarkii* J.Buchholz and N.E.Gray	-	-	Guyana (Pakaraima Mountains), Venezuela [Bolivar (Carrao-tepui, Uaipan-tepui, Cerro Jaua), Amazonas (Neblina Massif)]	LC
190	*Podocarpus subtropicalis* de Laub.	*Podocarpus subtropicalis* var. *medogensis*, *Podocarpus subtropicalis* subsp. *medogensis*	-	China (Sichuan, Yunnan)	DD
191	*Podocarpus sylvestris* J.Buchholz	*Podocarpus colliculatus*, *Podocarpus novae-caledoniae* var. *colliculatus*	-	New Caledonia (Grande Terre, Ile des Pins)	LC
192	*Podocarpus tepuiensis* J.Buchholz and N.E.Gray	-	-	Ecuador (Cordillera del Condo), Venezuela (Bolivar, Amazonas)	LC
193	*Podocarpus teysmannii* Miq.	*Nageia teysmannii*, *Podocarpus epiphyticus*, *Podocarpus neriifolius* var. *polyanthus*	Kalek rotan, Sikuju	Myanmar, Indonesia (Sumatera), Malaysia (Peninsular Malaysia, Sabah), Brunei Darussalam	NT
194	*Podocarpus thevetiifolius* Zipp. ex Blume	*Margbensonia thevetiifolia*, *Nageia thevetiifolia*, *Podocarpus polystachyus subsp. thevetiifolius*, *Podocarpus polystachyus var. thevetiifolius*	-	Papua New Guinea	NA
195	*Podocarpus totara* G.Benn. ex D.Don	*Nageia totara*, *Podocarpus totara* var. *waihoensis*, *Podocarpus totara* subsp. *waihoensis*	Totara	New Zealand (North Island and South Island)	LC
196	*Podocarpus totara* var. *waihoensis* Wardle	-	Totara, Westland totara	New Zealand (West Coast of the South Island)	NT
197	*Podocarpus transiens* (Pilg.) de Laub.	*Podocarpus lambertii* var. *transiens*, *Podocarpus transiens* var. *harleyi*, *Podocarpus transiens* subsp. *harleyi*	-	Brazil (Bahia, Goiás, Minas Gerais, Paraná, Santa Catarina)	EN
198	*Podocarpus trinitensis* J.Buchholz and N.E.Gray	-	-	Trinidad and Tobago (El Tucuche)	LC
199	*Podocarpus urbanii* Pilg.	-	Blue mountain yacca, Yacca	Jamaica (St. Andrew, Portland and St. Thomas within the Blue and John Crow Mountains)	CR
200	*Podocarpus vanuatuensis* de Laub.	-	-	Vanuatu	DD
201	*Podocarpus victorinianus* Carabia	*Podocarpus leonii*	-	Cuba	NE
202	*Prumnopitys andina* (Poepp. ex Endl.) de Laub.	*Nageia andina*, *Nageia valdiviana*, *Podocarpus andinus*, *Podocarpus spicatus*, *Podocarpus valdivianus*, *Prumnopitys andina* subsp. *blijdensteinii*, *Prumnopitys elegans*, *Prumnopitys spicata*, *Stachycarpus andinus*	Lleuque, Llleuqui, Uva de la Cordillera	Chile (Biobío, La Araucania, Maule), Argentina (Neuquen)	VU
203	*Prumnopitys taxifolia* (Sol. ex D.Don) de Laub.	*Dacrydium mai*, *Dacrydium taxifolium*, *Nageia spicata*, *Podocarpus spicatus*, *Stachycarpus spicatus*	Matai, Black pine	New Zealand (North Island and South Island)	VU
204	*Prumnopitys montana* (Humb. and Bonpl. ex Willd.) de Laub.	*Botryopitys densifolia*, *Botryopitys meridensis*, *Botryopitys montana*, *Dacrydium distichum*, *Nageia montana*, *Podocarpus curvifolius*, *Podocarpus humboldtii*, *Podocarpus montanus* var. *densifolius*, *Podocarpus montanus* var. *diversifolius*, *Podocarpus montanus* var. *meridensis*, *Podocarpus taxifolius*, *Podocarpus taxifolius var. communis*, *Podocarpus taxifolius var. densifolius*, *Stachycarpus meridensis*, *Stachycarpus taxifolius*, *Taxus montana*, *Torreya montana*	-	Bolivia (Cochabamba), Colombia (Belmira, San Andres, Arabuca, Villa de Leiva, Pensilvania, Cauca, Cesar, La Guajira, Magdalena, Quindío, Risarald, Tolima), Ecuado (Azuay, Cañar, Loja, Morona-Santiago, Zamora-Chinchipe), Peru (Cajamarca, Junín, Pasco, San Martín), Venezuela (Lara, Tachira, Zulia)	VU
205	*Pectinopitys exigua* (De Laub.) C.N.Page	-	Pino colorado, Jatun pino, Pino castilla, Pino negro	Bolivia (Cochabamba, Chuquisaca, Santa Cruz)	NT
206	*Pectinopitys ferruginea* (G.Benn. ex D.Don in Lamb.) C.N.Page	*Nageia ferruginea*, *Podocarpus ferrugineus*, *Stachycarpus ferrugineus*, *Stachypitys ferruginea*	Miro, Brown pine	New Zealand (North Island, South Island and Stewart Island)	LC
207	*Pectinopitys ferruginoides* (R.H.Compton) C.N.Page	*Podocarpus distichus*, *Podocarpus distichus* var. *maialis*, *Podocarpus ferruginoides*, *Stachycarpus distichus*, *Stachycarpus ferruginoides*, *Stachypitys disticha*, *Stachypitys ferruginoides*	-	New Caledonia	LC
208	*Pectinopitys harmsiana* (Pilg.) C.N.Page	*Podocarpus harmsianus*, *Podocarpus utilior*, *Prumnopitys utilior*	Uncumanu, Yellow miro	Bolivia (Abel Iturralde, Franz Tamayo, Sud Yungas), Colombia (Cauca, Quindío, Risaralda, Tolima, Sierra Nevada de Santa Marta), Ecuador (Loja), Peru (Ayacucho, Cajamarca, Cusco, Junín, Pasco, Piura, San Martín), Venezuela (Vargas, Miranda, Aragua, Yaracuy)	NT
209	*Pectinopitys ladei* (F.M.Bailey) C.N.Page	*Stachypitys ladei*, *Podocarpus ladei*, *Stachycarpus ladei*	Mount spurgeon black pine or Mount spurgeon brown pine	Australia (Queensland)	NT
210	*Pectinopitys standleyi* (J. Buchholz and N.E.Gray) C.N.Page	*Podocarpus standleyi*, *Stachycarpus standleyi*	Cipresillo, Ciprecillo, Ciprés lorito	Costa Rica (Alajuela, Cartago, Heredia, San José)	EN
211	*Retrophyllum comptonii* (J.Buchholz) C.N.Page	*Decussocarpus comptonii*, *Nageia comptonii*, *Podocarpus comptonii*	-	New Caledonia (Port Boise to Mt Ignambi)	LC
212	*Retrophyllum filicifolium* (N.E.Gray) R.R.Mill	*Podocarpus filicifolius*	Lehil, moegò	Indonesia (Maluku), Papua New Guinea (Bismarck Archipelago)	LC
213	*Retrophyllum minus* (Carrière) C.N.Page	*Nageia minor*, *Podocarpus minor*, *Podocarpus palustris*, *Decussocarpus minor*, *Retrophyllum minor*	Bois bouchon	New Caledonia (Grande Terre, Province Sud: Prony, Baie du Sud, Lac en Huit, Rivière des Lacs, Plaine des Lacs)	EN
214	*Retrophyllum piresii* (Silba) C.N.Page	*Decussocarpus piresii*, *Nageia piresii*	-	Brazil (Rondônia), Bolivia, Peru	DD
215	*Retrophyllum rospigliosii* (Pilg.) C.N.Page	*Decussocarpus rospigliosii*, *Nageia rospigliosii*, *Podocarpus rospigliosii*, *Torreya bogotensis*	Pino hayuelo, Diablo fuerte, Pino de monte, Pino real, Pino romero, Romerillo fino, Romerillo rojo, Saucecillo	Bolivia (Lap Paz), Colombia (Antioquia, Cundinamarca, Magdalena, Norte de Santander, Santander del Sur), Ecuador (Sucumbíos, Zamora-Chinchip), Peru (Junín, Pasco), Venezuela (Táchira, Mérida, Trujillo)	VU
216	*Retrophyllum vitiense* (Seem.) C.N.Page	*Decussocarpus vitiensis*, *Nageia vitiensis*, *Podocarpus vitiensis*	Ailumu, Dakua salusalu, Kau solo, Mungo	Fiji, Indonesia (Maluku, Papua), Papua New Guinea (Bismarck Archipelago), Solomon Islands (Santa Cruz Island), Vanuatu (Torba)	LC
217	*Saxegothaea conspicua* Lindl.	*Squamataxus albertiana*	Prince albert’s yew, Mañío, Mañío de hojas cortas, Mañío hembra, Mañío macho, Maniú	Argentina (Chubut, Neuquén, Rio Negro), Chile (Aisén, Biobío, La Araucania, Los Lagos, Maule)	NT
218	*Sundacarpus amarus* (Blume) C.N.Page	*Nageia amara*, *Nageia eurhyncha*, *Podocarpus amara*, *Podocarpus dulcamara*, *Podocarpus eurhyncha*, *Podocarpus pedunculatus*, *Prumnopitys amara*	Black pine, Choopoola	Australia (Queensland), Indonesia (Jawa, Maluku, Papua, Sulawesi, Sumatera), Malaysia (Sabah), Papua New Guinea (Bismarck Archipelago), Philippines, Timor-Leste	LC

**Table 2:** An updated checklist of podocarps fossil sp.ecies of extant genera.

S#	Fossil Taxa	Reported by	Part	Era/Period	Age Range Ma	Type Specimen	Distribution
1	*Acmopyle antarctica*	Florin, 1940 [27]	Leaf axis	Eocene	99.7 to 55.8	S132020-Swedish Museum of Natural History	Seymour Island (Antarctic Peninsula)
2	*Acmopyle compactus*	Pole, 1992 [28]	Leaf (Cuticle)	Middle–Late Eocene	48.6 to 33.9	S049-University of Tasmania	Hasties (Tasmania, Australia)
3	*Acmopyle engelhardti*	Florin, 1940 [27]	Leaf axis	Eocene–Miocene	55.8 to 48.6	PBDB 8673	(9 records) Rio Negro, Argentina
4	*Acmopyle florinii*	Hill and Carpenter, 1991 [29]	Sterile axis (Cuticle)	Late Paleocene	58.7 to 55.8	LB-063-University of Tasmania	Lake Bungarby (New South Wales, Australia)
5	*Acmopyle glabra*	Hill and Carpenter, 1991 [29]	Sterile axis (Cuticle)	Oligocene–Eocene	55.8 to 28.4	RPE-006-University of Tasmania	(9 records) Cethana—Oligocene, Regatta Point—Eocene (Tasmania, Australia)
6	*Acmopyle masonii*	Pole, 1997 [30]	Sterile axis (Cuticle)	Miocene	-	SB 1149-University of Tasmania.	Manuherikia Group, Central Otago, New Zealand
7	*Acmopyle setiger*	Hill and Carpenter, 1991; Carpenter and Pole, 1995; Townrow, 1965 [29,31,32]	Leaf (Cuticle)	Early Eocene	48.6 to 33.9	B-001-University of Tasmania	Lake Lefroy, Buckland and Monitor Bores (Tasmania and Western Australia)
8	*Acmopyle tasmanica*	Hill and Carpenter, 1991 [29]	Leaf (Cuticle)	Eocene	48.6 to 33.9	LA-060-University of Tasmania	Loch Aber (Tasmania, Australia)
9	*Dacrycarpus acutifolius*	Wells and Hill, 1989 [33]	Sterile axis (Cuticle)	Oligocene to Early Miocene	28.4 to 15.97	M-235- University of Tasmania	Monpeelyata (Tasmania, Australia)
10	*Dacrycarpus arcuatus*	Wells and Hill, 1989 [33]	Sterile axis (Cuticle)	Oligocene to Early Miocene	28.4 to 15.97	LRR1-243-University of Tasmania	Little Rapid River (Tasmania, Australia)
11	*Dacrycarpus carpenterii*	Jordan, 1995 [34]	Leaf	Early Pleistocene	2.588 to 0.126	RPU 525-University of Tasmania	Regatta Point (Tasmania, Australia)
12	*Dacrycarpus chilensis*	Wilf, 2012 [35]	Leaf (Cuticle)	Eocene		MMG PB SAT	Chile
13	*Dacrycarpus crenulatus*	Wells and Hill, 1989 [33]	Sterile axis (Cuticle)	Oligocene to Miocene	28.4 to 15.97	P-221-University of Tasmania	Pioneer—tin mine (Tasmania, Australia)
14	*Dacrycarpus cupressiformis*	Wells and Hill, 1989 [33]	Sterile axis (Cuticle)	Early Oligocene	33.9 to 23.0	LRR2-023-University of Tasmania	Little Rapid River 2 (Tasmania, Australia)
15	*Dacrycarpus dacrydoides*	Pole, 1992; 1997 [28,30]	Leaf (Cuticle)	Miocene	-	SB 1149-University of Tasmania.	Manuherikia Group, Central Otago, New Zealand
16	*Dacrycarpus geminus*	Pole, 1992 [28]	Leaf (Cuticle)	Eocene	56 to 33.9	S115-University of Tasmania	Hasties (Tasmania, Australia)
17	*Dacrycarpus guipingensis*	Wu et al., 2021 [36]	Seed cone+ Sterile axis	Miocene	-	GP109-Museum of Biology, Sun Yat-sen University	Guangxi, South China
18	*Dacrycarpus involutus*	Wells and Hill, 1989 [33]	Sterile axis (Cuticle)	Oligocene to Miocene	28.4 to 15.97	M2023-University of Tasmania	Monpeelyata (Tasmania, Australia)
19	*Dacrycarpus elandensis*	Hill and Whang, 2000 [37]	Pollen cone, leaf	Miocene	-	ELD-005-University of Adelaide	Elands, New South Wales
20	*Dacrycarpus falcatus*	Carpenter, 1991 [38]	Sterile axis (Cuticle)	Oligocene	35	C-052, 203, 619-University of Tasmania	Cethana (Tasmania, Australia)
21	*Dacrycarpus lanceolatus*	Wells and Hill, 1989 [33]	Sterile axis (Cuticle)	Oligocene to Miocene	28.4 to 15.97	M-1186-University of Tasmania	Monpeelyata (Tasmania, Australia)
22	*Dacrycarpus latrobensis*	Hill and Carpenter, 1991 [29]	Sterile axis (Cuticle)	Oligocene to Miocene	28.4 to 23.03	P 15714- Museum of Victoria, Melbourne	Southeastern Australia (Yallourn and Bacchus-3 locations)
23	*Dacrycarpus linearis*	Wells and Hill, 1989 [33]	Sterile axis (Cuticle)	Oligocene	28.4 to 23.03	LRR2-051-University of Tasmania	Little Rapid River 2 (Tasmania, Australia)
24	*Dacrycarpus linifolius*	Wells and Hill, 1989 [33]	Sterile axis (Cuticle)	Eocene to Oligocene	55.8 to 28.4	LRR1-851-University of Tasmania	Little Rapid River 1 (Tasmania, Australia) and Regatta Point (Tasmania, Australia)
25	*Dacrycarpus microfolius*	Jordan et al., 2011 [39]	Cuticle	Oligocene–Miocene	28.4 to 15.97	OU33024-Geology Museum (OU), University of Otago	F45/f0394, middle Gore Lignite Measure (Newvale Mine, New Zealand)
26	*Dacrycarpus mucronatus*	Wells and Hill, 1989; Carpenter, 1991; Lewis and Drinnan, 2013 [33,38,40]	Seed cone+ Sterile axis (Cuticle)	Eocene–Oligocene–Miocene	48.6 to 15.97	LRR2-044-University of Tasmania, (RPE-060-62, and RPE-4620	Little Rapid River 2 (Tasmania); Regatta Point (Tasmania) Cethana (Tasmania); Lochaber (Naracoorte, South Australia)
27	*Dacrycarpus patulus*	Hill and Merrifield, 1993 [41]	Leaf	Eocene–Oligocene	48.6 to 23.03	WAM P.84.34-Western Australian Museum	West Dale (Western Australia, Australia)
28	*Dacrycarpus praecupressinus*	Greenwood, 1987; Mill and Hill, 2004 [42,43]	Sterile axis (Leaves)	Eocene	37.2 to 33.9	F 51245-Geological Survey of New South Wales	Vegetable Creek—Witherdens Tunnel (NSW, Australia)
29	*Dacrycarpus puertae*	Wilf, 2012 [35]	Seed cone+ Sterile axis	Eocene	52–77.9	MPEF-Pb 4983-	Patagonia, Argentina
30	*Dacrycarpus* sp.	Pole et al., 1993 [44]	Leaf (Cuticle)	Late Oligocene–Early Miocene	-	SB282	Berwick Quarry, Victoria (Australia)
31	*Dacrycarpus* sp.	Carpenter and Pole, 1995 [32]	Leaf (Cuticle)	Middle Eocene	-	CD2999-University of Tasmania	Lefroy and Cowan Paleodrainages, Western Australia (Australia)
32	*Dacrycarpus* sp.	Carpenter, 1991 [38]	Foliage (Cuticle)	Oligocene	35	-	Cethana (Tasmania, Australia)
33	*Dacrycarpus* sp.	Carpenter et al., 1994 [45]	Foliage (Cuticle)	Oligocene–Early Miocene	-	R. J. Carpenter and R. S. Hill (unpublished data)	Lea River (Tasmania, Australia)
34	*Dacrydium aciculare*	Wells and Hill, 1989 [33]	Leaf (Cuticle)	Oligocene	33.9 to 28.4	LRR1-441-University of Tasmania	Little Rapid River 1 (Tasmania, Australia)
35	*Dacrydium fimbriatus*	Hill and Christophel, 2001 [46]	Sterile axis (Cuticle)	Middle Eocene	48.6 to 37.2	NC-004-University of Adelaide	Nelly Creek (South Australia, Australia)
36	*Dacrydium microphyllum*	Jordan et al., 2011 [39]	Cuticle	Oligocene–Miocene	28.4 to 15.97	OU33026-Geology Museum (OU), University of Otago	New Vale Mine, Waimumu (Coalfield, Southland, New Zealand)
37	*Dacrydium mucronatus*	Hill and Christophel, 2001 [46]	Fertile axis (Cuticle)	Eocene	48.6 to 37.2	NC-002- University of Adelaide	Nelly Creek (South Australia, Australia)
38	*Dacrydium rhomboideum*	Cookson and Pike, 1953; Blackburn, 1985 [47,48]	Foliage + Seeds	Oligocene–Miocene	28.4 to 15.97	P 209942	Morwell and Yallourm, Victoria (Australia)
39	*Dacrydium sinuosum*	Wells and Hill, 1989 [33]	Leaf (Cuticle)	Oligocene–Miocene	28.4 to 15.97	P-631-University of Tasmania	Pioneer- tin mine (Tasmania, Australia)
40	*Dacrydium tasmanicum*	Wells and Hill, 1989 [33]	Sterile axis (Cuticle)	Oligocene	33.9 to 28.4	LRR1-1031-University of Tasmania	Little Rapid River 1 (Tasmania, Australia)
41	*Dacrydium waimumuensis*	Jordan et al., 2011 [39]	Cuticle	Oligocene–Miocene	28.4 to 15.97	OU33025-Geology Museum (OU), University of Otago	F45/f0394, middle Gore Lignite Measure (Newvale Mine, New Zealand)
42	*Dacrydium* Sp1	Carpenter, 1991 [38]	Foliage	Early Oligocene	35	C-202, 471-University of Tasmania	Cethana (Tasmania, Australia)
43	*Dacrydium* Sp2	Carpenter, 1991 [38]	Foliage	Early Oligocene	35	C-517, 519-University of Tasmania	Cethana (Tasmania, Australia)
44	*Dacrydium* Sp1	Carpenter and Pole, 1995 [32]	Foliage (disp.ersed cuticles)	Middle Eocene	-	CD2999 and DWT495-University of Tasmania	Lefroy and cowan paleodrainage, Western Australia
45	*Dacrydium* Sp2	Carpenter and Pole, 1995 [32]	Foliage (disp.ersed cuticles)	Middle Eocene	-	CD2999-University of Tasmania	Lefroy and cowan paleodrainage, Western Australia
46	*Dacrydium* Sp	Blackburn, 1985 [48]	Foliage	Oligocene–Miocene	-	-	Morwell, Victoria (Australia)
47	*Dacrydium* Sp	Blackburn, 1985 [48]	Foliage	Oligocene–Miocene	-	-	Morwell, Victoria (Australia)
48	*Falcatifolium eocenica*	Hill and Scriven, 1999 [49]	Sterile axis (Cuticle)	Middle Eocene	37.2 to 33.9	2351 and 2350-State Herbarium of South Australia.	ALCOA Anglesea Site II coal mine (Victoria, Australia)
49	*Halocarpus highstedii*	Jordan et al., 2011 [39]	Cuticle	Oligocene–Miocene	28.4 to 15.97	OU32899-Geology Museum (OU), University of Otago	F45/f0394, middle Gore Lignite Measure (Newvale Mine, New Zealand)
50	*Lagarostrobos franklinii*	Wells and Hill, 1989; Hill and Macphail, 1985; Carpenter et al., 1994; Jordan, 1995; Jordan et al., 2011 [33,34,39,45,50]	Seed cones and foliage	Late Pliocene–Early Pleistocene	2.588 to 0.126	RPU-190-University of Tasmania	Regatta Point (Tasmania, Australia)
51	*Lagarostrobos marginatus*	Wells and Hill, 1989 [33]	Sterile axis (Cuticle)	Oligocene	33.9 to 28.4	LRR1-701-University of Tasmania	Little Rapid River 1 (Tasmania, Australia)
52	*Lagarostrobos* Sp	Peter, 1985 [51]	Sterile axis	Middle Cretaceous	145-100.5	-	Winton, Queensland
53	*Lagarostrobos colensoi* (correct name—*Manoao colensoi*)	Carpenter, 1991 [38]	Leaf (Cuticle)	Oligocene	35	-	Cethana (Tasmania, Australia)
54	*Lepidothamnus intermedius*	Pole, 1997 [30]	Sterile axis (Cuticle)	Miocene	-	S-632-University of Tasmania.	Manuherikia Group, Central Otago, New Zealand
55	*Lepidothamnus diemenensis*	Pole, 1992 [28]	Sterile axis (Cuticle)	Eocene	48.6 to 33.9	S014-University of Tasmania	Hasties (Tasmania, Australia)
56	*Lepidothamnus*	Peter, 1985 [51]	Seed cones and foliage	Middle Cretaceous	145-100.5	-	Winton, Queensland
57	*Microcachrys tetragona*	Jordan, 1995 [34]	Seed and sterile axes	Early Pleistocene		RPU2-University of Tasmania	Regatta Point (Tasmania, Australia)
58	*Microcachrys novaezelandiae*	Carpenter et al., 2011 [52]	Leaf (Cuticle)	Oligocene–Miocene	28.4 to 15.97	OU32896-Geology Museum (OU), University of Otago	F45/f0394, middle Gore Lignite Measure (Newvale Mine, New Zealand)
59	*Nageia hainanensis*	Jin et al., 2010 [53]	Leaf (Cuticle)	Eocene	-	CC-1200 a, b-The Museum of Biology of Sun Yat-sen University, Guangzhou, China	Changchang Basin, Hainan Island, south China
60	*Nageia maomingensis*	Liu et al., 2015 [54]	Leaf (Cuticle)	Late Eocene	-	MMJ1-001-The Museum of Biology, Sun Yat-sen University, Guangzhou, China.	Maoming Basin, Jintang, Maoming, Guangdong Province, South China.
61	(*Podocarpus*) *Nageia ryosekiensis*	Kimura et al., 1988 [55]	Leafy branches, connected seed, detached leaves	Lower Cretaceous	-	Makino Botanical Garden, Kochi	Southwest Japan
62	(*Podocarpus*) *Nageia sujfunensis*	Krassilov, 1965 [56]	Leaf (Cuticle)	Early Cretaceous	-	27/71-Far East Geological Institute, USSR Academy of Sciences	Far East Russia
63	*Pherosp.haera sommervillae* (Name correction *Microstrobos sommervillae*)	Townrow, 1965 [31]		Early Eocene	-	-	Buckland sediments in southeastern Tasmania
64	*Pherosp.haera microfolius* (Name correction *Microstrobos microfolius*)	Wells and Hill, 1989 [33]	Sterile axis (Cuticle)	Oligocene–Miocene	28.4 to 23.03	M-1155-University of Tasmania	Mudstone lens cutting Monpeelyata canal (Tasmania, Australia)
65	*Phyllocladus aberensis*	Hill, 1989 [57]	Leaf	Oligocene (Middle–Late Eocene)	28.4 to 23.03	LRR1-951-University of Tasmania	Little Rapid River 2 (Tasmania, Australia)
66	*Phyllocladus annulatus*	Hill, 1989 [57]	Leaf (Cuticle)	Oligocene	33.9 to 23.03	P-742-University of Tasmania	Pioneer (Tasmania, Australia)
67	*Phyllocladus aspleniifolius*	Ettingshausen, 1887, 1888; Cookson and Pike, 1954; Hill and Macphail, 1985; Hill, 1989, 1988; Pole, 1992 [28,50,57,58,59,60]	Leaf (Cuticle)	Late Eocene (Quaternary, Eocene and Cretaceous)	56-33.9 (84.9 to 0.012)	S 106-University of Tasmania	Deep leads NSW; Regatta point; Hasties, Tasmania (Australia) and Antarctica
68	*Phyllocladus elongatus*	Jordan et al., 2011 [39]	Leaf	Oligocene–Miocene	28.4 to 15.97	OU32901-Geology Museum (OU), University of Otago	F45/f0394, middle Gore Lignite Measure (Newvale Mine, New Zealand)
69	*Phyllocladus lobatus*	Hill, 1989 [57]	Cuticle	Oligocene	28.4 to 23.03	LRR1-1649- University of Tasmania	Little Rapid River 2 (Tasmania, Australia)
70	*Phyllocladus morwellensis*	Deane, 1925; Cookson and Pike, 1954; Hill, 1989 [57,60,61]	Leaf	Oligocene	28.4 to 15.97	P-15873-Museum of Victoria	Near Morwell, (Victoria, Australia)
71	*Phyllocladus palmeri*	Pole and Moore, 2011 [62]	Leaf (Cuticle)	Late Miocene	6 to 6.5	AU P340a and b-School of geography, geology and environmental science, University of Auckland	Near Matuora, (Coromandel Peninsula, New Zealand)
72	*Phyllocladus* Sp (*P*. *lobatus*)	Carpenter, 1991 [38]	Leaf (Cuticle)	Oligocene	-	-	Cethana, (Tasmania, Australia)
73	*Phyllocladus* Sp2 (*P*. *hypophyllus*)	Carpenter, 1991 [38]	Leaf (Cuticle)	Oligocene	-	-	Cethana, (Tasmania, Australia)
74	*Phyllocladus* Sp	Mc Loughlin and Hill, 1996; Mc Loughlin et al., 2001 [63,64]	Leaf (Cuticle)	Late Eocene	-	-	Kojonup, Western Australia
75	*Phyllocladus* Sp	Pole, 1992 [28]	Leaf (Cuticle)	Late Miocene	-	-	Near Cromwell, (South Island, New Zealand)
76	*Phyllocladus* Sp	Pole, 1992 [28]	Leaf (Cuticle)	Miocene	-	OU30068-Department of Geology, University of Otago.	Manuherikia Group, Central Otago, New Zealand
77	*Phyllocladus* Sp	Liz Kennedy, 2020 (unpublished)	Seed cones (*Phyllocladus toatoa*)	Miocene	23 to 5.3	-	Coromandel, North Island, New Zealand
78	*Podocarpus andiniformis (Subgenus Foliatus)*	Wilf et al., 2017/Berry, 1922 [65,66]	Leaf (Cuticle)	Late Triassic and Early Eocene	-	-	Patagonia, Argentina
79	*Podocarpus alwyniae* (*Subgenus Podocarpus*)	Pole, 1992 [28]	Sterile axis (Cuticle)	Miocene	-	OU29708, H411fD45-Department of Geology, University of Otago.	Manuherikia Group, Central Otago, New Zealand
80	*Podocarpus oligocenicus*	Awasthi et al., 1992 [67]	Leaf (Cuticle)	Oligocene	-	-	Mizoram, India; Manipur, India
81	*Podocarpus araucoensis* (as *Decussocarpus araucoensis*)	Berry, 1922; Mill and Hill, 2004 [43,66]	Foliage (Cuticle)	Eocene	55.8 to 33.9	-	Chile
82	*Podocarpus brownei* (probably *Retrophyllum* or *Falcatifolium*)	Greenwood, 1987 (*Decussocarpus brownei*); Mill and Hill, 2004 [42,43]	Foliage (Cuticle)	Eocene	37.2 to 33.9	2345-State Herbarium of South Australia	ALCOA Anglesea Site II coal mine (Victoria, Australia)
83	*Podocarpus fildesensis*	Zhou and Li, 1994 [68]	Foliage	Cretaceous	84.9 to 66.043	-	Half Three Point assemblage (Antarctica)
84	*Podocarpus inopinatus*	Florin, 1940 [27]	Foliage	Paleogene, Eocene–Miocene	55.8 to 33.9	-	Chile
85	*Podocarpus platyphyllum*	Greenwood, 1987 [42]	Leaf (Cuticle)	Middle to Late Eocene	37.2 to 33.9	1816-State Herbarium of South Australia	ALCOA Anglesea Site II coal mine (Victoria, Australia)
86	*Podocarpus sinuatus*	Pole, 1992 [28]	Leaf (Cuticle)	Eocene	48.6 to 33.9	S112-University of Tasmania	Hasties (Tasmania, Australia)
87	*Podocarpus pliomacrophyllus* (Subgenus *Foliatus*)	Chen et al., 2019; Wu et al., 2021 [69,70]	Leaf (Cuticle)	Lower Pliocene	-	MBU-16395- Institute of Palaeontology and Stratigraphy, Lanzhou University, Gansu Province, China.	Mannong Village (western Yunnan, China); Tuantian Town, Yunnan Province, southwestern China
88	*Podocarpus travisiae (Subgenus Podocarpus)*	Pole, 1993 [71]	Leaf	Miocene	23.03 to 15.97	0U30780- Department of Geology, University of Otago	Foulden Hills (New Zealand)
89	*Podocarpus yunnanensis (Subgenus Foliatus)*	Wu et al., 2021 [70]	Leaf (Cuticle)	Early Pliocene	-	MBU-19122301-Institute of Palaeontology and Stratigraphy, Lanzhou University	Tuantian Town, Yunnan Province, southwestern China
90	*Podocarpus forrestii (Subgenus Foliatus)*	Wu et al., 2021 [70]	Leaf (Cuticle)	Early Pliocene	-	MBU-20191221-Institute of Palaeontology and Stratigraphy, Lanzhou University	Tuantian Town, Yunnan Province, southwestern China
91	*Podocarpus tasmanicus (Subgenus Podocarpus)*	Townrow, 1965 [31]	Leaf (Cuticle)	Eocene	-	81905-University of Tasmania	Bed of Tea Tree Rivulet, Buckland, Tasmania, Australia
92	*Podocarpus strzeleckianus (Subgenus Podocarpus)*	Townrow, 1965 [31]	Leaf (Cuticle)	Eocene	-	81917-University of Tasmania	Bed of Tea Tree Rivulet, Buckland, Tasmania, Australia
93	*Podocarpus witherdenensis* (Subgenus Podocarpus)	Hill and Carpenter, 1991 [29]	Fertile axis+ seed cones (Cuticle)	Eocene	37.2 to 33.9	MMF 1201-Geological Survey of New South Wales, Sydney	Vegetable Creek—Witherdens Tunnel (NSW, Australia)
94	*Podocarpus* Sp	Carpenter et al., 1994 [45]	Foliage (Cuticle)	Oligocene–Early Miocene	-	R. J. Carpenter and R. S. Hill (unpublished data)	Lea River (Tasmania, Australia)
95	*Podocarpus* Sp1	Carpenter, 1991 [38]	Foliage (Cuticle)	Oligocene	-	University of Tasmania	Cethana, (Tasmania, Australia)
96	*Podocarpus* Sp2	Carpenter, 1991 [38]	Foliage (Cuticle)	Oligocene	-	C-251, 274, 275, 342, 492- University of Tasmania	Cethana, (Tasmania, Australia)
97	*Podocarpus* Sp	Carpenter et al., 1994 [45]	Foliage (Cuticle)	Oligocene–Early Miocene	-	R. S. Hill (unpublished data)	Little Rapid River (Tasmania, Australia)
98	*Podocarpus* Sp	Hill and Macphail, 1985 [50]	Foliage (Cuticle)	Late Pliocene–Early Pleistocene	-	University of Tasmania	Regatta Point (Tasmania, Australia)
99	*Podocarpus* Sp (Subgenus Podocarpus)	Jordan et al., 2011 [39]	Foliage (Cuticle)	Late Oligocene–Early Miocene	-	OU33027- Geology Museum, University of Otago	Newvale site, (South Island, New Zealand)
100	*Podocarpus Sp*	Pole, 1997 [72]	Foliage (Cuticle)	Miocene	-	H41/f74, S788-University of Tasmania.	Manuherikia Group, Central Otago, New Zealand
101	*Podocarpus Sp*	He and Wang, 2021 [73]	Leaf (Cuticle)	Miocene	17 to 14	-	Guangchang County, Jiangxi Province, southeastern China
102	*Retrophyllum australe*	Hill and Merrifield, 1993 [41]	Sterile axis	Eocene–Oligocene	48.6 to 23.03	WAM P.88.96-Western Australian Museum	West Dale (Western Australia, Australia)
103	*Retrophyllum superstes*	Wilf et al., 2017 [65]	Sterile axis (Leafy twig)	Cretaceous–Paleocene	70.6 to 61.7	MPEF-Pb 8910- Patagonia, Argentina	LefE (Chubut, Argentina)
104	*Retrophyllum oxyphyllum (Retrophyllum sp.iralifolium*)	Wilf, 2020 [74]	Sterile axis, cuticle, leaves, fertile axis	Eocene	52	MLP-4234 and MPEF–Pb 8915a Museo Paleontológico Egidio Feruglio	Trelew, Argentina
105	*Retrophyllum vulcanense*	Pole, 1992 [28]	Sterile axis (Cuticle)	Miocene	-	OU29857-Department of Geology, University of Otago.	Manuherikia Group, Central Otago, New Zealand
106	*Prumnopitys tasmanica*	Mill and Hill, 2004; Greenwood, 1987 [42,43]	Sterile axis (Cuticle)	Eocene	-	81905-University of Tasmania	Alcoa Anglesea, Victoria, Australia
107	*Prumnopitys montana*	Pole, 1992 [28]	Cuticle	Eocene	48.6 to 33.9	S 110.-University of Tasmania	Hasties (Tasmania, Australia)
108	*Prumnopitys opihiensis*	Pole, 1997 [72]	Cuticle	Cretaceous/Eocene	99.7 to 48.6	OU30932- University of Otago	Taratu Formation (New Zealand)
109	*Prumnopitys portensis*	Pole, 1992 [28]	Leaf	Eocene	48.6 to 33.9	S056-University of Tasmania	Hasties (Tasmania, Australia)
110	*Prumnopitys taxifolia* (Leaf morphology is similar to that of *Sundacarpus*)	Pole, 1997 [30]	Leaf	Miocene	-	SB 1154--Department of Geology, University of Otago.	Manuherikia Group, Central Otago, New Zealand
111	*Sundacarpus anglica*	Page, 2019 [75]	Leaf (Cuticle)	Eocene	48.6 to 33.9	V.46883- Natural History Museum, BM	Bandulska (Bournemouth, England)
112	*Sundacarpus tzagajanicus*	Page, 2019 [75]	Leaf (Cuticle)	Uppermost Cretaceous (Earliest Paleocene—65.5–61.7 Ma)	65.5–61.7	575-126-Far Eastern Scientific Centre, Vladivostok	Bureya River (Russia)

**Table 3:** A brief historical overview of major taxonomic classifications of Podocarpaceae (Type genus *Podocarpus elongatus*).

Taxonomist	Taxonomic Treatment
Endlicher, 1847 [98]	He classified Podocarpaceae into three genera i. *Podocarpus* (with four sections i. *Eupodocarpus*, ii. *Stachycarpus*, iii. *Nageia* and iv. *Dacrycarpus*), 2. *Dacrydium* Sol. ex G. Forst, 3. *Microcachrys* Hook. f.
Pilger, 1926 [99]	He considered Podocarpaceae as subfamilies Podocarpoideae with Subgenus I. Protopodocarpus (with section i. *Eupodocarpus*, ii. *Dacrycarpus*), II. Stachycarpus with section B. i. *Nageia* ii. *Saxegothaea* iii. Microcachrys iv. *Pherosphaera* v. *Acmopyle* vi. *Dacrydium*, vii. section A, viii. *Microcarpus* and Phyllocladoideae with i. *Phyllocladus*
Buchholz and Gray, 1948 [100,101]	Classified *Podocarpus* into nine sections (*P*. sect. *Eupodocarpus*, *P*. sect. *Nageia*, *P*. sect. *Afrocarpus*, *P*. sect. *Polypodiopsis*, *P*. sect. *Microcarpus*, *P*. sect. *Dacrycarpus*, *P*. sect. *Sundacarpus*, *P*. sect. *Stachycarpus*)
Keng, 1973 [102]	Divided into two families, i.e., Podocarpaceae and Phyllocladaceae
Gaussen, 1974 [103]	Raised this group into suborder Podocarpineae and divided into three families, i.e., Podocarpaceae, Phyllocladaceae and Saxegothaeaceae.
de Laubenfels, 1985 [104]	Classified *Podocarpus* into two subgenera and 18 sections (subgenus *Podocarpus*: sect. Podocarpus, sect. Scytopodium, sect. capitulatis, sect. Australis, sect. Crassiforms, sect. Pratensis, sect. Lanceolatis, sect. Pumilis, sect. Nemoralis, subgenus *Foliolatus*: sect. Globulus, sect. Foliolatus, sect. Acuminatis, sect. Longifoliolatus, sect. Gracilis, sect. Macrostachyus, sect. Spinulosus, sect. Rumphius, sect. Polystachyus.)
Quinn, 1987 [105]	Placed back *Phyllocladus* in Podocarpaceae
Hart, 1987 [90]	Recognized 15 genera *Lagarostrobos*, *Microstrobos* (*Pherosphaera*), *Microcachrys*, *Lepidothamnus*, *Halocarpus*, *Parasitaxus*, *Dacrycarpus*, *Falcatifolium*, *Dacrydium*, *Acmopyle*, *Nageia*, *Saxegothaea*, *Phyllocladus*, *Prumnopitys* and *Podocarpus*
Page, 1988 [106]	Recognized eight genera in s.l. *Podocarpus* and five in *Dacrydium*
Page, 1990 [107]	Classified Podocarpaceae into *Acmopyle*, *Falcatifolium*, *Dacrydium*, *Halocarpus*, *Lagarostrobos*, *Lepidothamnus*, *Microcachrys*, *Microstrobos* (*Pherosphaera*), *Phyllocladus* and *Podocarpus* (*P*. subg. *Podocarpus* and *P*. subg. *Foliolatus*) *Nageia* (*N*. sect. *Nageia*, *N*. sect. *Afrocarpus*, *N*. sect. *Polypodiopsis*), *Dacrycarpus*, *Parasitaxus*, *Prumnopitys*, *Sundacarpus*, *Saxegothaea*
Dezhi, 1992 [108]	Placed *Nageia* into a new family Nageiaceae
Kelch, 1998 [7]	Produced the phylogeny of Podocarpaceae-based molecular markers (18S RNA) of 10 genera in the following sequences: *Podocarpus*, *Dacrycarpus*, *Pherosphaera*, *Microcachrys*, *Afrocarpus*, *Saxegothaea*, *Dacrydium*, *Parasitaxus*, *Lagarostrobos* and *Phyllocladus*.
Conran et al., 2000 [93]	Produced the phylogeny of Podocarpaceae-based molecular markers (*rbcL*) of 16 genera in the following sequences: *Afrocarpus*, *Nageia*, *Retrophyllum*, *Podocarpus*, *Dacrydium*, *Falcatifolium*, *Dacrycarpus*, *Acmopyle*, *Pherosphaera*, *Microcachrys*, *Lagarostrobos*, *Manoao*, *Prumnopitys*, *Halocarpus*, *Phyllocladus*, *Lepidothamnus* and *Saxegothaea*.
Kelch, 2002 [14]	Produced the phylogeny of Podocarpaceae-based molecular markers (18S RNA) of 16 genera in the following sequences: *Dacrydium*, *Falcatifolium*, *Dacrycarpus*, *Pherosphaera*, *Microcachrys*, *Saxegothaea*, *Acmopyle*, *Nageia*, *Afrocarpus*, *Podocarpus*, *Lagarostrobos*, *Halocarpus*, *Parasitaxus*, *Phyllocladus*, *Lepidothamnus* and *Prumnopitys*.
Sinclair et al., 2002 [94]	Constructed the phylogeny of 18 genera-based molecular markers (trnL-trnF+ITS2) in the following sequences: *Afrocarpus*, *Nageia*, *Retrophyllum*, *Podocarpus*, *Dacrydium*, *Falcatifolium*, *Dacrycarpus*, *Acmopyle*, *Pherosphaera*, *Microcachrys*, *Saxegothaea*, *Lagarostrobos*, *Manoao*, *Parasitaxus*, *Halocarpus*, *Prumnopitys*, *Lepidothamnus* and *Phyllocladus*.
Wagstaff, 2004 [83]	Constructed the phylogeny of 9 genera-based molecular markers (*rbcL*+*matK*) in the following sequences: *Afrocarpus*, *Podocarpus*, *Dacrydium*, *Saxegothaea*, *Halocarpus*, *Lepidothamnus*, *Prumnopitys* and *Phyllocladus*.
Biffin et al., 2012 [8]	Constructed the phylogeny of 18 genera based molecular markers (*matK*+ trnL-trnF+ITS2) in the following sequences: *Afrocarpus*, *Nageia*, *Retrophyllum*, *Podocarpus*, *Dacrydium*, *Falcatifolium*, *Dacrycarpus*, *Acmopyle*, *Pherosphaera*, *Microcachrys*, *Saxegothaea*, *Lagarostrobos*, *Manoao*, *Parasitaxus*, *Halocarpus*, *Prumnopitys*, *Lepidothamnus* and *Phyllocladus*.
Knopf et al., 2012 [92]	Constructed the phylogeny of 18 genera-based molecular markers (ITS1+NEEDLY intron 2+ anatomy and morphology) in the following sequences: *Afrocarpus*, *Nageia*, *Retrophyllum*, *Podocarpus*, *Dacrydium*, *Falcatifolium*, *Dacrycarpus*, *Pherosphaera*, *Microcachrys*, *Halocarpus*, *Lepidothamnus*, *Lagarostrobos*, *Manoao*, *Phyllocladus*, *Prumnopitys* and *Saxegothaea*.
Little et al., 2013 [95]	Used DNA barcoding (*matK*, *rbcL* and nrITS2 DNA barcodes) for the identification of Podocarpaceae (18 genera and 145 species) and to construct the phylogenetic tree
Lu et al., 2014 [11]	Constructed the phylogeny of 18 genera-based molecular markers (*LEAFY*+NEEDLY CDS+ introns) in the following sequences: *Afrocarpus*, *Nageia*, *Retrophyllum*, *Podocarpus*, *Dacrydium*, *Falcatifolium*, *Dacrycarpus*, *Acmopyle*, *Pherosphaera*, *Saxegothaea*, *Microcachrys*, *Lagarostrobos*, *Manoao*, *Parasitaxus*, *Phyllocladus*, *Lepidothamnus*, *Halocarpus* and *Prumnopitys*.
Contreras et al., 2017 [109]	Constructed the phylogeny of 18 genera-based molecular markers in the following sequences: *Afrocarpus*, *Nageia*, *Retrophyllum*, *Podocarpus*, *Dacrydium*, *Falcatifolium*, *Dacrycarpus*, *Acmopyle*, *Pherosphaera*, *Microcachrys*, *Saxegothaea*, *Halocarpus*, *Phyllocladus*, *Lepidothamnus*, *Prumnopitys*, *Lagarostrobos*, *Manoao* and *Parasitaxus*.
Leslie et al., 2018 [12]	Recently constructed the phylogeny of 19 genera-based molecular markers (18S, *rbcL* and *matK*) in the following sequences: *Podocarpus*, *Afrocarpus*, *Nageia*, *Retrophyllum*, *Falcatifolium*, *Dacrydium*, *Dacrycarpus*, *Acmopyle*, *Pherosphaera*, *Microcachrys*, *Saxegothaea*, *Prumnopitys*, *Sundacarpus*, *Manoao*, *Lagarostrobos*, *Parasitaxus*, *Halocarpus*, *Phyllocladus* and *Lepidothamnus*.
Sudianto et al., 2019 [110]	Constructed the phylogeny tree of 12 genera based on Plastome in the following sequences: *Afrocarpus*, *Nageia*, *Retrophyllum*, *Podocarpus*, *Dacrycarpus*, *Dacrydium*, *Pherosphaera*, *Saxegothaea*, *Phyllocladus*, *Lagarostrobos*, *Lepidothamnus* and *Prumnopitys*.
Page, 2019 [75]	Recently divided the genus *Prumnopitys* into two genera, *Prumnopitys* (Subgenus Prumnopitys and Subgenus Botryopitys) and *Pectinopitys*.
Khan et al., 2023 [current classification]	*Dacrycarpus*, *Halocarpus*, *Lepidothamnus*, *Manoao*, *Dacrydium*, *Lagarostrobos*, *Microcachrys*, *Pherosphaera*, *Parasitaxus*, *Acmopyle*, *Falcatifolium*, *Phyllocladus*, *Retrophyllum*, *Prumnopitys*, *Pectinopitys*, *Afrocarpus*, *Nageia*, *Podocarpus*, *Sundacarpus* and *Saxegothaea*.

## Data Availability

Data available in article supplementary material. Additional supporting information may be found in the online version of the article at the publisher’s website.

## Supplementary Materials

The following supporting information can be downloaded at: https://www.mdpi.com/article/10.3390/plants12051171/s1. Figure S1. *Phyllocladus aspleniifolius* (with phylloclades) found in rainforest Tasmania. Figure S2. Leaf dimorphism in *Dacrycarpus dacrydioides*. Table S1. Fossil taxa used for calibration of the phylogeny [165,166].
