# Supplementary material for: Diversity, Distribution, Systematics and Conservation Status of Podocarpaceae
Source: Plants (Basel). 2023 Mar 3;12(5):1171. doi: 10.3390/plants12051171 (PMC10005643; doi:10.3390/plants12051171)
Supplement: Supplementary file 1 [file plants-12-01171-s001.zip › Supplementary File.pdf]

Table S1. Fossil taxa used for calibration of the phylogeny.

S#	Fossil taxa	Period	Minimum Age Constraint (Ma)	Reference
1	*Acmopyle florinii*	Late Paleocene	57	Hill and Carpenter, 1991
2	*Nageia hainanensis*	Eocene	50	Liu et al., 2015
3	*Podocarpus witherdenensis*	Eocene	37.2	Hill and Carpenter, 1991
4	*Dacrycarpus puertae*	Eocene	60	Wilf et al., 2012
5	*Dacrydium rhomboideum*	Oligocene-Miocene	28.4	Cookson and pike, 1953; Blackburn, 1985
6	*Falcatifolium eocenica*	Middle Eocene	37.2	Hill and Scriven, 1999
7	*Pherosphaera microfolius*	Oligocene-Miocene	28.4	Wells and Hill, 1989
8	*Microcachrys novaezelandiae*	Oligocene-Miocene	28.4	Carpenter et al., 2011
9	*Halocarpus highstedii*	Oligocene-Miocene	28.4	Jordan et al., 2011
10	*Lagarostrobos marginatus*	Oligocene	34	Wells and Hill, 1989
11	*Manoao colensoi*	Oligocene	35	Carpenter, 1991
12	*Prumnopitys opihiensis*	Cretaceous/Eocene	60	Pole, 1997
13	*Lepidothamnus*	Middle Cretaceous	120	Peter, 1985
14	*Phyllocladus annulatus*	Oligocene	33	Hill, 1989
15	*Retrophyllum oxyphyllum*	Eocene	50	Wilf, 2020
16	*Sundacarpus anglica*	Eocene	48.6	Page, 2019
17	*Austrohamia minuta*	Lower Jurassic	160	Bodnar and Escapa, 2016
18	*Agathis vittata*	Eocene	55	Hill et al., 2008
19	*Araucaria*	Early Jurassic	190	Axsmith et al., 2008
20	Podocarp	Permian	255	Blomenkemper et al., 2018

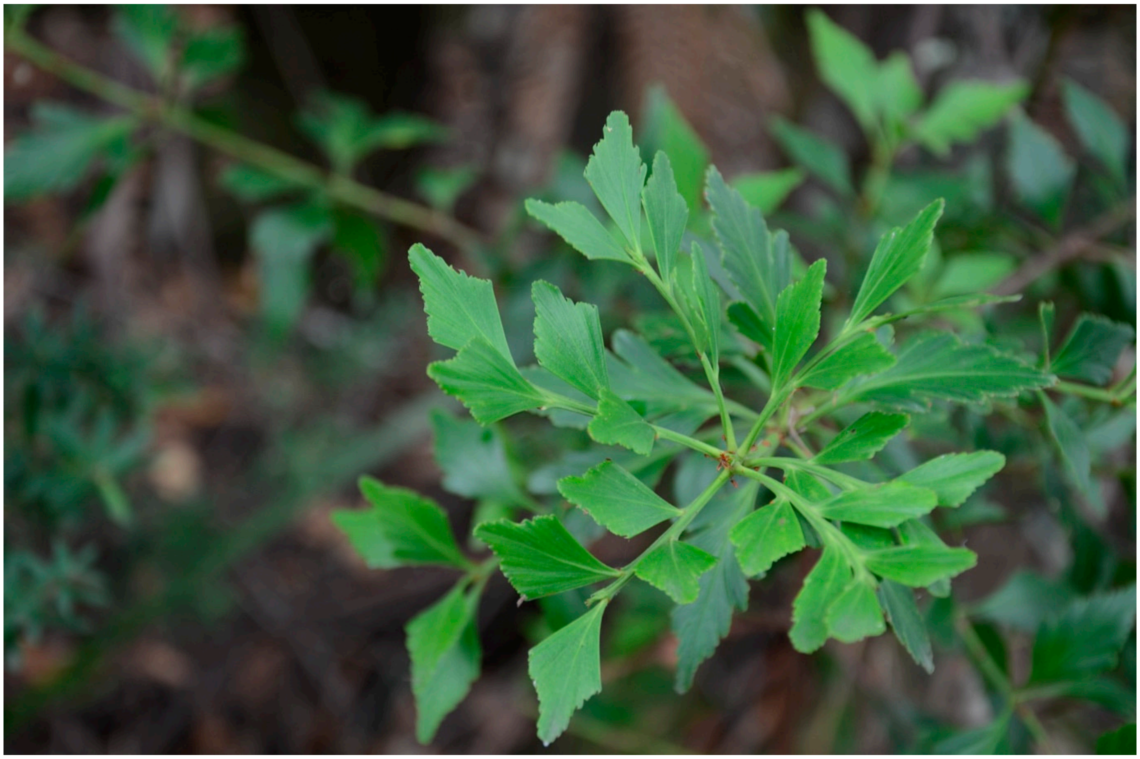

Figure S1. *Phyllocladus aspleniifolius* (with phylloclades) found in rainforest Tasmania.

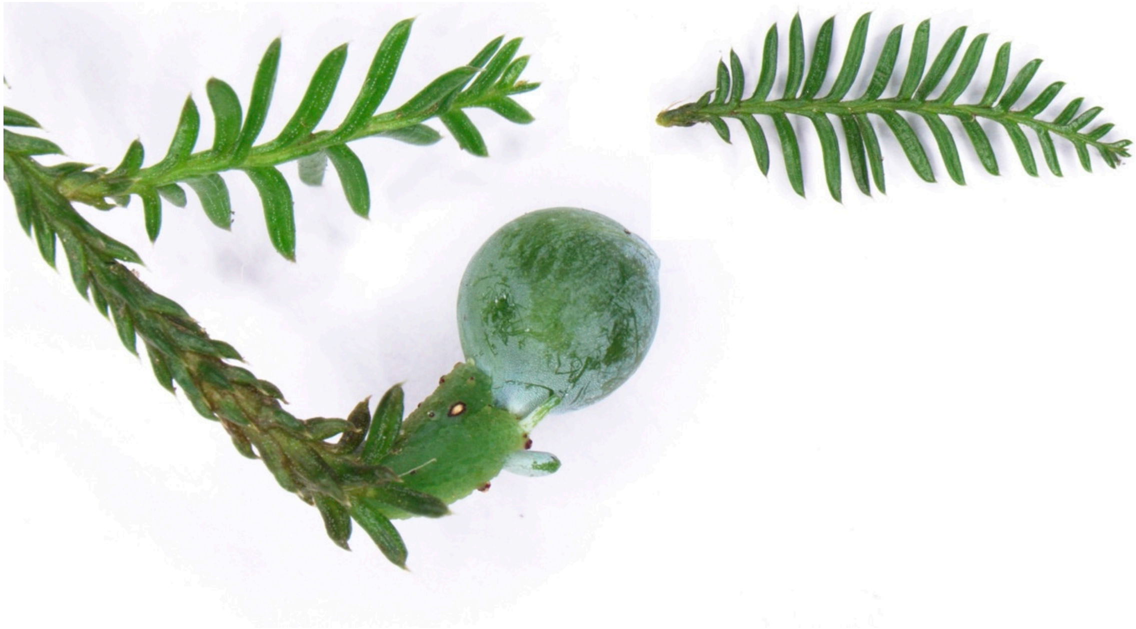

Figure S2. Leaf dimorphism in *Dacrycarpus dacrydioides*.
